# G Protein‐Coupled Receptor Signaling: Implications and Therapeutic Development Advances in Cancers

**DOI:** 10.1002/mco2.70375

**Published:** 2025-09-16

**Authors:** Inamu Rashid Khan, Sana Khurshid, Saud Almawash, Rakesh Kumar, Ammira S. Al‐Shabeeb Akil, Ajaz A. Bhat, Muzafar A. Macha

**Affiliations:** ^1^ Department of Zoology Central University of Kashmir Ganderbal Kashmir India; ^2^ Watson‐Crick Centre for Molecular Medicine Islamic University of Science and Technology Kashmir India; ^3^ Department of Pharmaceutics College of Pharmacy Shaqra University Shaqra Saudi Arabia; ^4^ School of Biotechnology Shri Mata Vaishno Devi University Katra Jammu and Kashmir India; ^5^ Metabolic and Mendelian Disorders Clinical Research Program Precision OMICs Research & Translational Science Sidra Medicine Doha Qatar

**Keywords:** GPCRs, signaling, diseases, cancer, bias, therapeutics

## Abstract

G protein‐coupled receptors (GPCRs) are the largest and most diverse class of membrane proteins, mediating cellular responses to a wide range of extracellular stimuli. GPCRs initiate complex intracellular signaling networks that regulate vital physiological functions and are associated with numerous diseases, including various types of cancer. Their conserved seven‐transmembrane (7TM) structure enables these signaling networks by allowing interactions with multiple ligands and intracellular effectors. In several types of tumors, abnormal GPCR signaling promotes carcinogenesis by supporting immune evasion, cell proliferation, and therapeutic resistance. A significant research gap exists in fully understanding the molecular mechanisms behind pathway‐specific activation and biased ligand discovery of GPCRs, which could lead to the development of more effective therapies. This review examines the complexity of GPCRs, with a focus on their role in signaling through the differential activation of pathways regulated by β‐arrestin and G proteins. It discusses how targeted modulation of signaling outcomes by receptor mutants might offer therapeutic benefits in cancer treatment. The review also highlights emerging technologies, such as aptamers, PROTACs, and nanobodies, that more precisely target GPCRs. In addition to exploring receptor structure–function relationships and pathway selectivity, this review provides valuable insights into GPCR‐biased signaling and its implications in cancer biology.

## Graphical abstract

G protein‐coupled receptors (GPCRs) are the dynamic protein family in the human genome. GPCR dysregulation aids in the advancement of cancer through increased migration, survival, and proliferation. Novel treatments that target GPCR signaling include orthosteric ligands, biased receptor mutants, PROTACs, and antibody–drug conjugates. Biased ligands offer improved treatment selectivity and reduced side effects by preferentially activating one pathway over another.

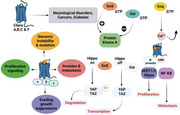

## 1 Introduction

The most extensive and diverse protein family encoded by the human genome is the G protein‐coupled receptors (GPCRs). They are found on the cell membrane and are essential for processing extracellular signals into vital biological responses [1]. GPCRs have evolved from a seven‐transmembrane (7TM) receptor with a common ancestor, resulting in a diverse superfamily of membrane receptors [2]. These receptors are encoded by around 1000 genes in the human genome, ranking them among the most prominent protein families [3, 4]. GPCRs are categorized into different classes based on similarities in the peptide chain patterns within the characteristic 7TM helical domains—the rhodopsin class (class A), secretin class (class B), adhesion class/glutamate class (class C), and frizzled/taste class (class F). The amino acid sequences of the human GPCR family serve to classify these subfamilies [5, 6]. The distinct structural features and ligand‐binding specificities in each GPCR subfamily are closely associated with their respective physiological functions [7].

GPCRs are selectively stabilized in specific conformations by biased ligands, which either stimulate or inhibit one or more alternative signaling pathways [8]. These ligands act on a broad spectrum of receptors, including adrenergic, dopaminergic, muscarinic, chemokine, and opioid receptors (ORs), each of which contributes to distinct physiological and pathological processes. A biased receptor refers to a genetically modified or mutant version of a receptor that, unlike the wild‐type (WT) receptor, can adopt a specific active conformation after ligand binding, selectively triggering a particular signaling pathway over others [9]. Among the key intracellular regulators of these signaling pathways are arrestins. Whereas β‐arrestins are widely distributed and act on a range of GPCRs, β‐arrestin 1 and 2 share around 80% sequence similarity and play overlapping and distinct functions in GPCR regulation [10, 11]. These are cytosolic adaptor proteins expressed in all cell types. Initially identified for their involvement in suppressing GPCR signaling by blocking contact with heterotrimeric G proteins, their roles have been discovered to go well beyond this inhibitory activity. β‐Arrestins block active GPCRs, facilitate receptor endocytosis, and activate downstream kinases, leading to the activation of specialized signaling pathways [12], which in turn elicit various physiological and cellular responses [12]. GPCRs play a pivotal role in cancer development by stimulating cell proliferation, survival, and migration in response to locally produced or circulating agonists. These agonists include chemokines, hormones, growth factors, and bioactive lipids, all of which contribute to critical tumor‐promoting processes such as angiogenesis, immune evasion, metastasis, and chronic inflammation [13, 14, 15]. Aberrant GPCR signaling has been observed in various cancers, resulting from receptor overexpression, constitutive activation, or excessive agonist production, and is associated with a poor prognosis and resistance to therapy. Upon agonist binding, GPCRs undergo rapid conformational changes that activate heterotrimeric G proteins, composed of Gα, Gβ, and Gγ subunits. The activated Gα subunit and Gβγ dimer modulate diverse intracellular effectors and second messengers, leading to altered gene expression and cellular behavior. In parallel, activated receptors are phosphorylated by GPCR kinases, which in turn recruit β‐arrestins. These adaptor proteins not only mediate receptor desensitization and internalization but also scaffold mitogenic signaling cascades such as mitogen‐activated protein kinase (MAPK)/ERK, PI3K/AKT, and JNK pathways, all of which are implicated in cancer progression [4, 16]. The pharmacological modulation of GPCR activity through orthosteric ligands—molecules that compete with endogenous ligands at the receptor's active site—offers a promising therapeutic strategy. Orthosteric agonists mimic the effects of natural ligands and can activate GPCR signaling, while antagonists block receptor activation by preventing ligand binding, thereby suppressing downstream oncogenic signaling. In some cases, inverse agonists can further inhibit constitutively active GPCRs by stabilizing their inactive conformations, reducing basal activity. This competitive binding mechanism enables precise control over aberrant GPCR activity, making GPCRs attractive drug targets for the treatment of multiple malignancies, including breast, prostate, ovarian, and gastrointestinal cancers [17, 18].

GPCRs are the targets of approximately 34% of US Food and Drug Administration (US FDA)‐approved medications, and the number of modulators in preclinical or clinical trials is increasing exponentially [19, 20, 21]. Ligands that preferentially activate one signaling route over another are thought to be biased and may improve therapeutic efficacy and safety [22, 23, 24]. Ligands that primarily activate G protein pathways enhance therapeutic outcomes by selectively activating G protein‐mediated signaling pathways while reducing or preventing β‐arrestin signaling. For instance, these biased ligands are engineered to specifically target G protein signaling at ORs, improving treatment efficacy [25, 26]. Various genetically biased altered D2 dopamine receptors, β‐arrestin‐biased mutants (A135R and M140D), and Gαi/o protein‐biased mutants (L125N and Y133L) have been extensively studied by predicting and modifying key residues crucial for interactions with G proteins or β‐arrestins, thereby revealing their distinct signaling pathways and functional properties [27]. In targeted protein degradation, proteolysis‐targeting chimeras (PROTACs) have provided an innovative approach to modify disease‐relevant GPCRs in cancers [28]. Recent advancements are enabling the creation of antibody–drug conjugates (ADCs) that target GPCRs, thereby expanding therapeutic options and facilitating more accurate tumor cell targeting [29, 30, 31, 32]. Collectively, the emerging modalities—nanobodies, aptamers, and gene therapy‐are expanding the therapeutic landscape for GPCR‐targeted interventions, offering new avenues for precision medicine [33, 34].

This review highlights the role of GPCRs in biased signaling networks, with a focus on their structure, signaling pathways, and therapeutic potential. It emphasizes the importance of signaling selectivity for drug development and explains how ligands can preferentially activate specific pathways, such as those mediated by β‐arrestins or G proteins. To enhance the understanding of the cellular mechanisms underlying biased signaling, the review discusses recent advances in structural biology, including the elucidation of GPCR structures and their complexes. It also examines the opportunities and challenges associated with translating these discoveries into more targeted and effective clinical therapies.

## 2 Classes of GPCRs, General Overview

**FIGURE 1:**
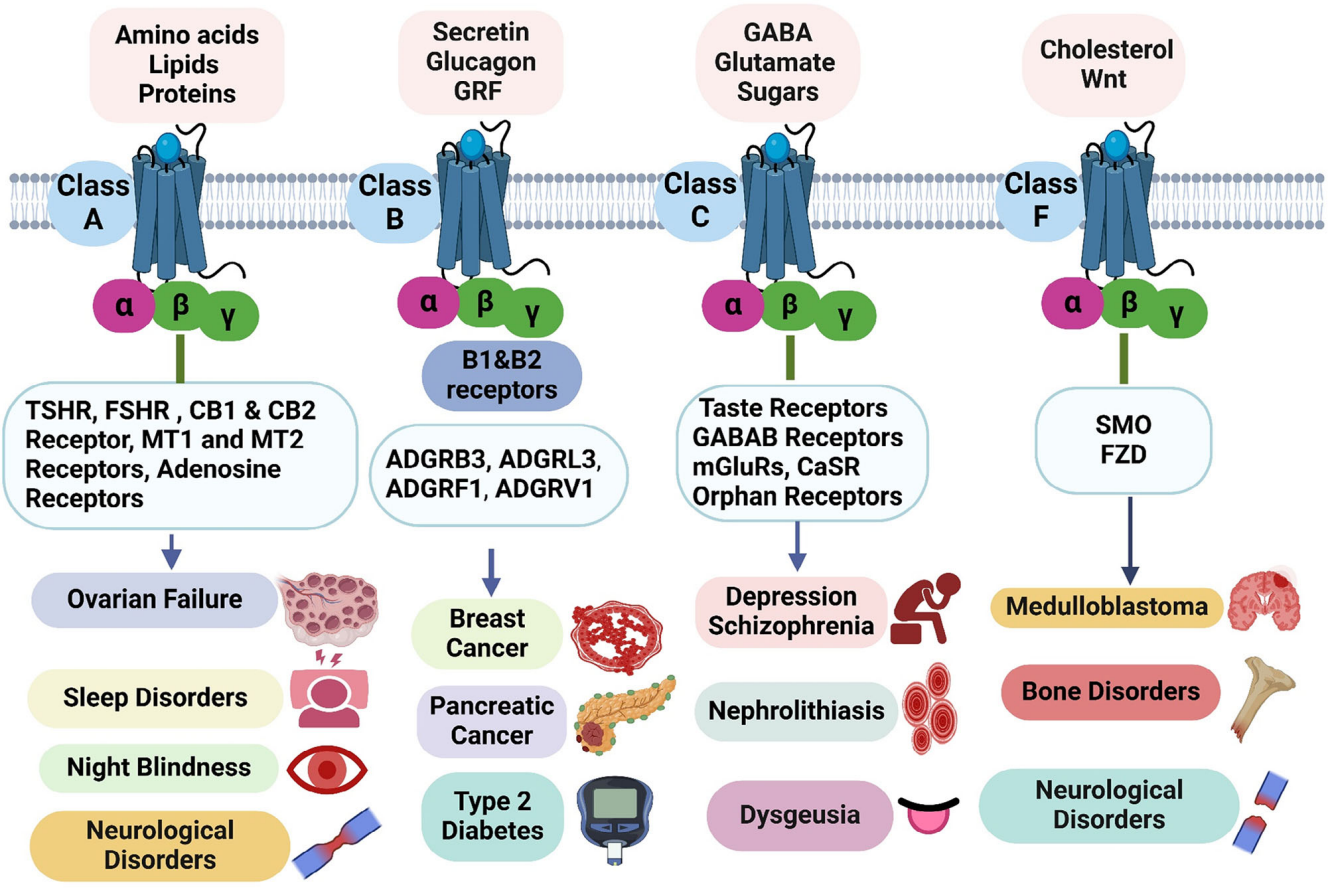
Diverse classes of GPCRs and their role in disease progression. This figure illustrates the classification of GPCRs and their critical roles in disease mechanisms. It emphasizes the relationship between ligand binding and the activation of specific signaling pathways, showcasing how each GPCR class uniquely contributes to pathological processes. The visual highlights the complexity of GPCR‐mediated responses and their significance as potential therapeutic targets, focusing on the interplay between ligand specificity and signaling diversity in various conditions. *Abbreviations*: GPCR—G protein‐coupled receptor, TSHR—thyroid‐stimulating hormone receptor, FSHR—follicle‐stimulating hormone receptor, CB1—cannabinoid receptor type 1, CB2—cannabinoid receptor type 2, MT1—melatonin receptor 1, MT2—melatonin receptor 2, ADGRB3—adhesion G protein‐coupled receptor B3, ADGRL3—adhesion G protein‐coupled receptor L3, GABA—gamma‐aminobutyric acid, mGluRs—metabotropic glutamate receptors, CaSR—calcium‐sensing receptor, SMO—smoothened receptor, FZ—frizzled receptor.

The various classes of GPCRs emphasize the distinct roles of each class in cellular signaling. It also highlights the diseases associated with these receptors, showcasing their relevance in health and pathology (Figure 1). This comprehensive overview underscores the potential of targeting specific GPCR classes in therapeutic interventions.

### 2.1 Class A GPCRs

Class A GPCRs are categorized into subgroups based on their specific ligand interactions. These subgroups include receptors for peptides, proteins, lipids, melatonin, nucleotides, steroids, sensory stimuli, and olfactory signals [19]. Not all class A GPCRs are considered orphan receptors. While class A GPCRs encompass a diverse range of receptors, only a subset remains “orphaned,” meaning their endogenous ligands have not yet been identified [35]. The intracellular domain's downstream effector proteins are activated when orthosteric ligands connect with the binding pocket created by the 7TM helices [36]. Many class A GPCRs have distinct ligand‐binding characteristics and activation mechanisms. A notable group is the glycoprotein hormone receptor subfamily, which includes essential receptors like FSHR, LHR, and TSHR, playing crucial roles in hormonal regulation and signaling [37, 38, 39]. Class A GPCRs comprise the odorant receptor (OR) and trace amine‐associated receptor families, two of the three categories of olfactory receptors that detect odorants. Olfactory sensory neurons were found to express specific molecules in extranasal tissues. These molecules play roles in various biological processes, indicating their potential as therapeutic and diagnostic targets [40]. Orphan GPCRs are a subset of class A GPCRs, where the “orphan” designation refers to the lack of a known ligand. Orphan GPCRs play a crucial role in regulating physiological processes and are linked to various human disorders. These include Alzheimer's disease, schizophrenia, type 2 diabetes, ADHD, cognitive decline, and different brain abnormalities, highlighting their clinical significance [41]. There are now about twelve structures known for orphan receptors. For instance, the ECL2 region functions as an inherent agonist for receptor activation [42, 43]. The development and metastasis of ovarian cancer are influenced by a large number of GPCRs and their particular ligands. Therefore, it would be highly beneficial to develop new chemotherapeutics to fully characterize the roles of these GPCRs during carcinogenesis [44]. Research on orphan GPCRs is still in its infancy, but promising opportunities emerge as more receptor structures are elucidated and detailed studies of constitutive activity and active conformations advance [45].

### 2.2 Class B GPCRs

Class B GPCRs are categorized into B1 secretin receptors and B2 adhesion receptors. Class B1 GPCRs undergo a sequence of conserved conformational changes within their 7‐transmembrane domain. The extracellular parts of TM6, TM7, and ECL3 move outward, while the extracellular part of TM1 moves inward, generating a V‐shaped pocket that enhances ligand binding [46]. The structural architecture of B1 receptors is comparable to their transmembrane domain, which has seven helices, and extracellular domain, which has 120–160 residues. These domains collaborate to facilitate ligand recognition [47]. The B1 receptor family activates various G protein subtypes to trigger downstream signaling, with Gs being the predominant subtype [48, 49]. These receptors are crucial for regulating several physiological functions, including blood sugar regulation, calcium homeostasis, and the motility and secretion of the gastrointestinal system [50, 51, 52]. The B2 adhesion GPCR (aGPCR) family includes 33 unique members, playing key roles in cellular signaling and communication. These receptors are involved in essential physiological functions, including endocrine regulation, reproduction, and cerebrovascular function. Mutations or abnormal expression in these receptors are associated with several diseases, including cancers and neurodevelopmental disorders [53].

### 2.3 Class C GPCRs

Class C GPCRs are classified into four groups according to the particular agonists they interact with: orphan receptors, amino acid receptors (such as γ‐aminobutyric acid receptors GABAB1/GABAB2, metabotropic glutamate receptors [mGluRs]), and taste receptors [54]. The calcium‐sensing receptor (CaSR), which predominantly couples with Gq, is a unique member of its subfamily [55]. The parathyroid glands and renal tubules are the primary sites of expression for the CaSR. It is essential for controlling the release of parathyroid hormone in response to blood calcium levels. Due to these actions, CaSR is considered a possible candidate gene for calcium nephrolithiasis [56]. Two members of the GABAB subfamily, GB1 and GB2, function as a heterodimer. GB1 binds the agonist, while GB2 plays a crucial role in coupling with the G protein heterotrimer [57, 58]. Both preclinical and clinical evidence suggest that the GABAB receptor holds significant potential as a therapeutic target for treating anxiety and depression. Identifying new GABAB receptor allosteric modulators and expanding knowledge of how specific intracellular GABAB receptor‐associated proteins influence GABAB receptor activity could lead to the development of GABAB receptor therapies for managing such disorders [59]. Taste GPCRs are in specialized taste receptor cells within taste buds. Type II taste GPCRs, such as monomeric bitter receptors and kokumi/CaSRs, are distinguished by their unique structure. On the other hand, type I taste GPCRs form heterodimeric complexes that function as receptors for sweet (TAS1R2/TAS1R3) and umami (TAS1R1/TAS1R3) tastes [60]. Activation of mGluR3 has been shown to have neuroprotective effects in astrocytes, while mGluR5 appears to exacerbate neurotoxicity associated with Aβ42 [61].

### 2.4 Class F GPCRs

Class F GPCRs differ from others in that they have a conserved 7TM domain linked to the N‐terminal cysteine‐rich domain (CRD) [62]. The CRD plays a crucial role in receptor activation and initiating downstream signaling, making it a key target for drug development [63]. Class F GPCRs encompass the smoothened (SMO) and frizzled (FZ) family receptors. Based on their different sensitivities for Wnt ligands and sequence similarities, the 10 members of the FZ receptor family are categorized into five subfamilies: FZ1/2/7, FZ3/6, FZ4, FZ5/8, and FZ9/10. The potential of these receptors as therapeutic targets is underscored by the fact that their alterations have been associated with several malignancies [64, 65, 66, 67]. Cancer, fibrosis, and neurodegeneration are among the many illnesses linked to FZs and their related Hedgehog (Hh) and Wnt signals [19]. SMO tends to have a straight TM6, but FZs show a kinked TM6 when activated. These different activation pathways may offer insights into targeting class F receptors to develop medications with enhanced pharmacological properties and selectivity [68]. SMO is integral to the Hh signaling pathway, playing a vital role in tissue repair and maintaining homeostasis [69]. In the activated state of SMO, compared with its inactive form, the intracellular end of TM6 shifts outward while TM5 moves inward [70]. The triggered SMO structure exhibits TM6 moving outward within the cell, reminiscent of structural changes in class A and B GPCRs [71].

In summary, GPCR classification into classes A, B, C, and F highlights their structural diversity and functional roles in key physiological processes, including neurotransmission, metabolism, and immune regulation. Dysregulation of these receptors contributes to cancer development, reinforcing their significance as therapeutic targets. A deeper understanding of class‐specific features can aid in designing targeted, biased ligands for more effective cancer therapies.

## 3 Signaling Role of Heterotrimeric G Proteins in Cellular Physiology

**FIGURE 2:**
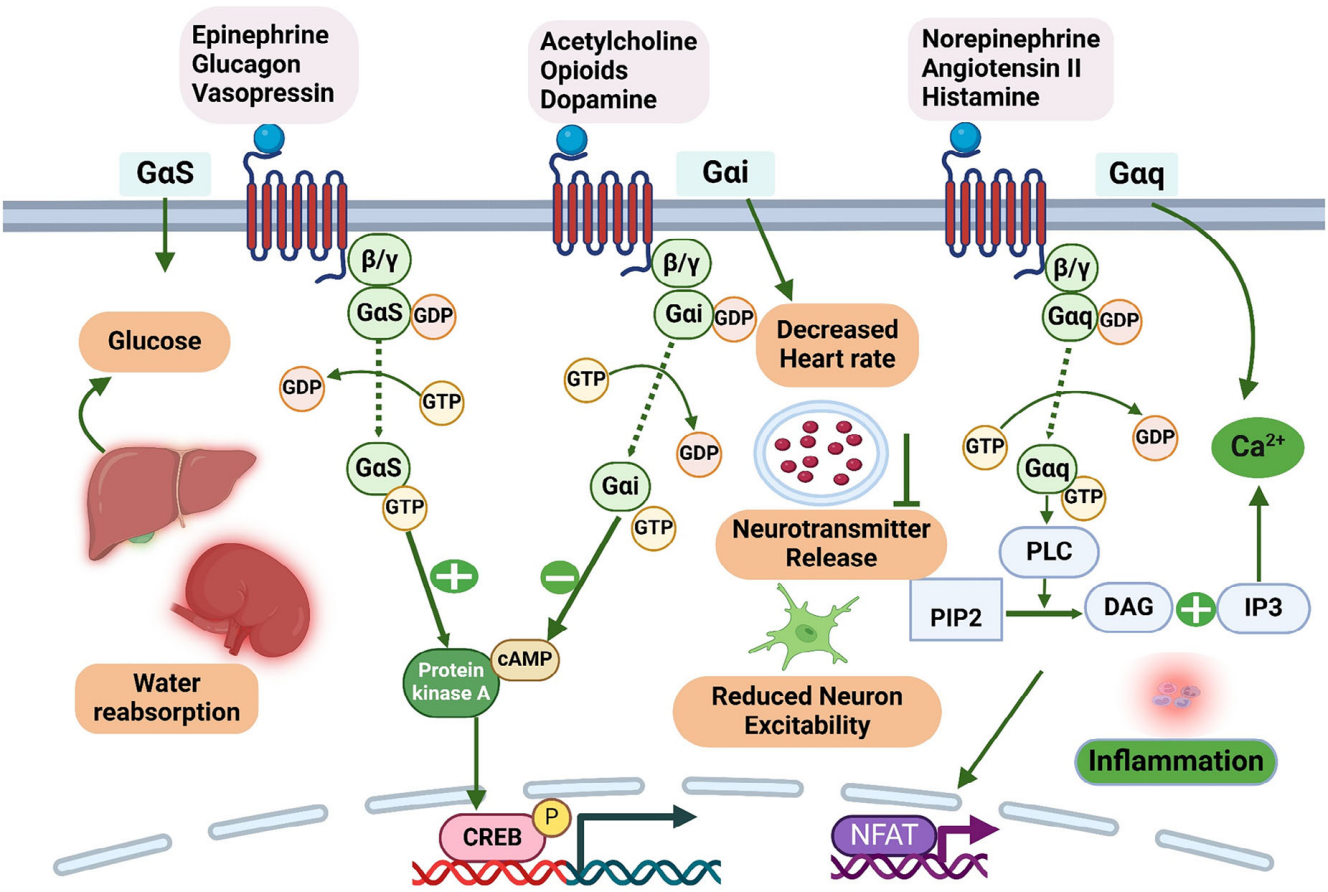
Distinct signaling branches of Gα proteins in GPCR pathways. The figure depicts the distinct signaling branches of Gαs, Gαq, and Gαi. This visual representation underscores the signaling associated with physiological impacts. Each branch highlights the subtle interplay between receptor activation and downstream effects. Understanding these signaling dynamics is crucial for harnessing GPCRs in therapeutic applications. *Abbreviations*: Gαs—stimulatory G protein alpha subunit, Gαi—inhibitory G protein alpha subunit, Gαq—G protein alpha q subunit, cAMP—cyclic adenosine monophosphate, PKA—protein kinase A, CREB—cAMP response element‐binding protein, PLC—phospholipase C, PIP2—phosphatidylinositol 4,5‐bisphosphate, DAG—diacylglycerol, IP3—inositol trisphosphate, NFAT—nuclear factor of activated T‐cells.

The G protein subtypes Gαq, Gαs, and Gαi illustrate their unique roles in cellular communication and showcases the variable signaling effects of each subtype. Gα, Gβ, and Gγ are the three subunits that make up the heterotrimeric G proteins, the primary signaling transducers in GPCR pathways [72]. Gαs subunits activate protein kinase A (PKA) and other targets by stimulating AC to convert ATP to cyclic adenosine monophosphate (cAMP). PKA phosphorylates a variety of effectors in the heart, including voltage‐gated L‐type Ca2+ channels, troponin I, myosin‐binding protein‐C, phospholamban, and the cardiac RYR2 (ryanodine receptor) [73]. Together, these subcellular elements are coordinated by βAR stimulation to enhance myofilament cross‐bridge cycling, thereby increasing both the relaxation rate (lusitropy) and the force of contraction (inotropy). Ca2+‐induced Ca2+ release occurs when action potential‐triggered L‐type Ca2+ channels initiate an inward Ca2+ entry, causing the RYR2 to release a considerable quantity of intracellular Ca2+. The RYR2 creates a Ca2+ spark, the primary intracellular Ca2+ source needed to develop and contract myofilament cross‐bridges. βAR activation affects the sinoatrial node ion channels, transporters, and hyperpolarization‐activated cyclic nucleotide‐gated channels to modulate heart rate (chronotropy) [74]. Gαq/11 catalyzes the conversion of membrane‐bound phosphatidylinositol 4,5‐bisphosphate to diacylglycerol and inositol 1,4,5‐trisphosphate (IP3) by activating phospholipase‐Cβ. The sarcoplasmic reticulum and nuclear envelope include intracellular Ca2+ release channels called cardiomyocyte IP3 receptors, which are activated by IP3. Calcineurin, a Ca2+ and calmodulin‐dependent serine/threonine protein phosphatase, and a nuclear pool of Ca2+‐calmodulin‐dependent protein kinase II, promote cardiac hypertrophic signaling. GPCR activation triggers IP3 production, which leads to Ca2+ release from the nuclear envelope through IP3 receptors, further contributing to the signaling process [75, 76]. The sarcoplasmic reticulum Ca2+ATPase pump that controls protein phospholamban is dephosphorylated when diacylglycerol and intracellular Ca2+ activate protein kinase C‐alpha (PKC‐α), which controls cardiac contractility by upregulating type 1 protein phosphatase [77]. Normal cardiac function depends on heterotrimeric G protein signaling; however, chronic, prolonged activation, especially of Gαs and Gαq/11, can lead to unfavorable ventricular remodeling and reduced cardiac performance. The detrimental effects of excessive G protein signaling on the heart include tachycardia and an increased risk of heart failure [78]. Gαi activation may help block Gαs signaling and have an inhibitory effect on AC activity. Therefore, following GPCR stimulation, negative feedback loops are essential for preserving cellular homeostasis. Regulators of G protein signaling (RGS) proteins act as GTPase‐activating proteins (GAPs), inhibiting GTP hydrolysis to Gα–GDP. By preventing GTP hydrolysis, these regulators stabilize the active Gα–GTP form [79]. This leads to reforming the heterotrimeric G protein complex, effectively terminating Gα‐mediated signaling (Figure 2).

## 4 GPCR Signaling Bias

Biased signaling refers to the process in which GPCRs can adopt numerous active conformations, and different ligands can stabilize unique receptor states, leading to the selective activation of specific downstream signaling pathways [80]. In contrast to the conventional perspective, which holds that all pathways are activated with equal efficacy, biased signaling permits the preferential activation of one pathway (such as the G protein‐mediated pathway) over another (such as the arrestin‐mediated pathway) [81, 82]. The arrestins play crucial roles at the receptor level, including desensitization, trafficking, and signaling [83]. When specific ligands preferentially induce β‐arrestin recruitment and signaling over G protein activation, the idea of β‐arrestin‐biased signaling emerges. These β‐arrestin‐biased agonists can simultaneously promote β‐arrestin‐dependent pathways and inhibit G protein signaling, resulting in distinct cellular and physiological consequences [84]. While β‐arrestin‐biased ligands primarily regulate the conformational states of helix 7, it has been demonstrated that unbiased ligands bind to the receptor, primarily shifting the equilibrium toward the G protein‐specific active state of helix 6. This indicated that, contrary to what was previously thought, phosphorylation of the receptor itself does not always result in a switching of the receptor coupling to different G proteins [85, 86]. β‐Arrestins 1 and 2 serve distinct cellular roles. Their differential nucleocytoplasmic shuttling arises from the location of the nuclear export sequence, which is present on the C‐terminus of β‐arrestin 2 and the N‐terminus of β‐arrestin 1 and 2, underscoring their unique regulatory mechanisms [87]. Numerous conformational states of β‐arrestin: receptor complexes are made possible by extra interactions with the intracellular loops and core of active receptors. By preventing further G protein activation, β‐arrestins bind to receptors, blocking interactions with the Gα subunit and leading to receptor desensitization [88, 89]. As signaling transducers and multipurpose scaffold proteins, β‐arrestins are associated with various outcomes and are essential for controlling other cellular effects and intracellular signal transmission and amplification. Accordingly, they have diverse roles in the control and development of cancerous tumors via many signaling pathways, for example, MAPK and PI3K/AKT [90, 91]. It has been demonstrated that β‐arrestin 1 serves as a framework for cytoskeleton changes that affect the motility of tumor cells [92, 93, 94]. Various researchers have demonstrated that nuclear β‐arrestin 1 can ensemble transcriptional responses to environmental disturbances, indicating new functions for β‐arrestin 1 in cancer progression [95, 96, 97]. A recent clinical investigation suggested that β‐arrestin 2 is a potential target for novel therapeutic methods and a significant predictive factor in advanced ovarian cancer [98]. Similarly, a recent study using breast cancer cells showed that downregulating the expression of β‐arrestin 1 and β‐arrestin 2 tended to enhance cell invasion and proliferation, while upregulating their expression levels inhibited these processes [99]. By suppressing androgen receptor (AR) signaling, β‐arrestin 2 in prostate cancer prevents cell viability and proliferation [100, 101]. However, other research supports the notion that β‐arrestin 2 activity contributes to the growth of human malignancies; β‐arrestin 2 is highly expressed in several human cancers, such as renal cell carcinoma and breast cancer, and is associated with a poor patient survival rate [102, 103, 104]. To summarize, β‐arrestins initiate intracellular signaling cascades without the need for G protein support by combining intrinsic cellular pathways with GPCR signals. This combination enables the development of novel treatments targeting β‐arrestin‐mediated pathways, a phenomenon called arrestin‐biased agonism [22]. To comprehend the mechanics behind the role of β‐arrestins in cancer, further research should be conducted on the anticancer and tumor suppressor efficacy of β‐arrestin isoforms, which will reveal their functional specialization.

## 5 Mutational Activation of GPCR Signaling in Cancer

**FIGURE 3:**
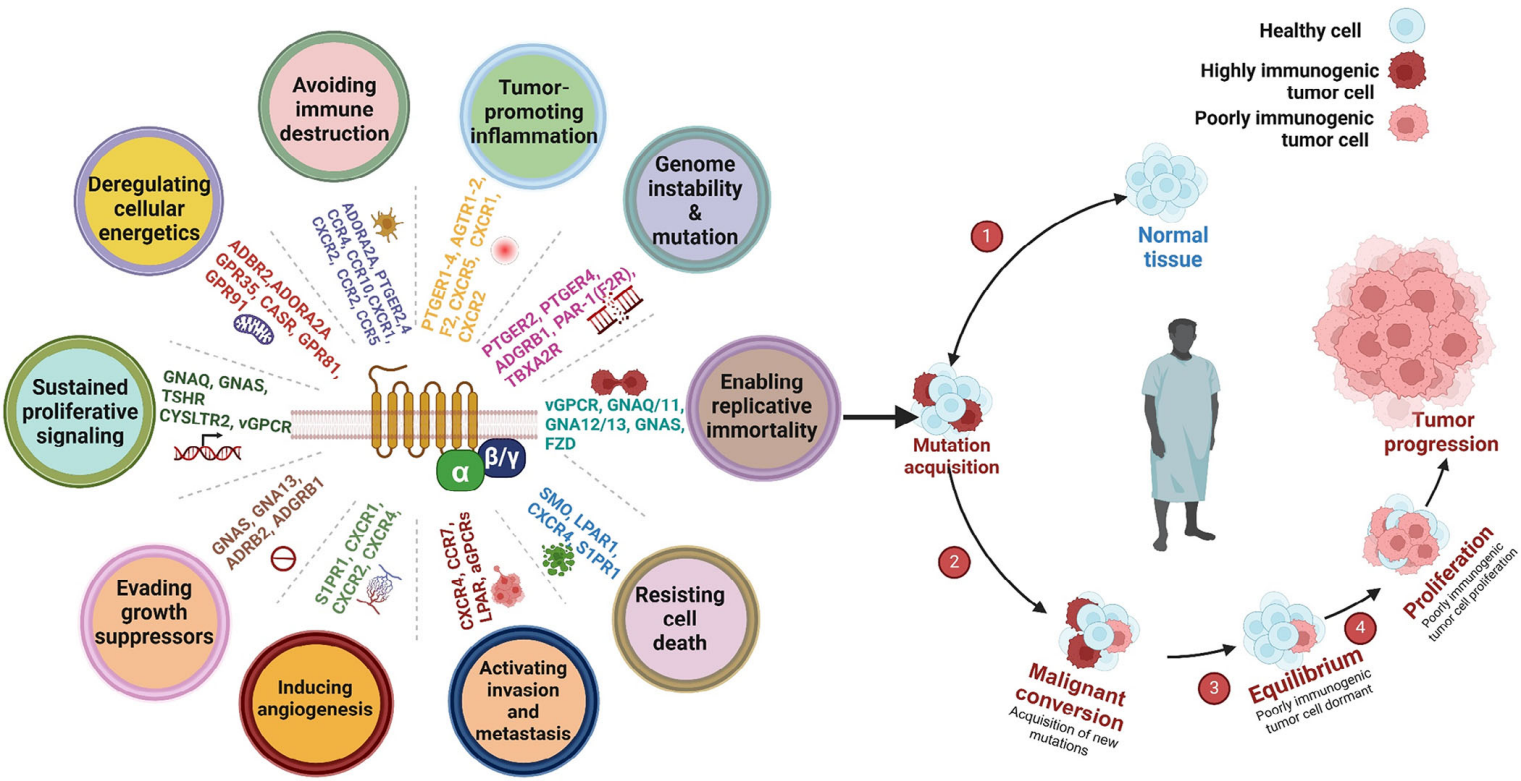
Roles of G proteins and GPCRs in cancer progression. Key G proteins and G protein‐coupled receptors (GPCRs) are highlighted as drivers of hallmark traits in cancer. Dysregulated signaling through these receptors supports tumor growth and progression by altering cellular processes and oncocrine networks within the tumor microenvironment. *Abbreviations*: GNAQ—G protein subunit alpha q, GNAS—G protein subunit alpha s, GNA12—G protein subunit alpha 12, GNA13—G protein subunit alpha 13, GNAI2—G protein subunit alpha i2, CXCR4—C‐X‐C chemokine receptor type 4, FZ7—frizzled class receptor 7, PAR1—protease‐activated receptor 1, VIPR2—vasoactive intestinal peptide receptor 2, P2Y2—purinergic receptor P2Y2, GPR68—G protein‐coupled receptor 68, LPAR1—lysophosphatidic acid receptor 1.

GPCRs and cellular transformation were first found to be directly related after the identification of the receptor for angiotensin (1–7) (MAS oncogene) in 1986 [14]. The GPCR protein family has since been linked to tumor growth and metastasis by molecular genetics, identifying significant GPCRs, their mutations, or altered expressions. Research has demonstrated that when a locally produced or circulating agonist activates GPCRs, it contributes to the growth of cancer cells. These agonists play a key role in inflammation‐related cancer, metastasis, and angiogenesis. Prior research has also examined the distinct GPCRs involved in cancer's functional activities, expression, and signaling [13, 14, 15]. A standard set of GPCRs was shown to be expressed by a particular type of cancer cell or tumor. Table 1 presents specific instances of a broad class of GPCRs that are highly expressed in various metastatic and primary tumor cells and are linked to the proliferation of tumor cells when triggered by circulating or locally generated ligands. Both cancer cells and the different cell types that comprise the tumor microenvironment (TME) exhibit high levels of GPCR expression. These receptors are crucial for mediating intercellular communication, a vital mechanism that influences the progression of tumors and cancer. Chemokine receptors stand out among the many GPCR families due to their role in cancer biology. These receptors can stimulate the growth of cancer cells, improve survival by preventing programmed cell death, and promote other characteristics of malignancy, including angiogenesis, invasion, and metastasis, by interacting with their ligands (Figure 3). They also serve as targets for unraveling tumor‐supportive connections in the microenvironment and as indicators of tumor behavior.

**TABLE 1:** Selected G protein‐coupled receptors, ligands, and signaling pathways involved in cancer.

Receptors	Ligands	Pathways	Cancer type	References
Protease‐activated receptors (PAR1 & 2) LPA	Thrombin, trypsin, or TFLLRN (PAR1) or SLIGKV (PAR2) Lysophosphatidic acid (Gαq)	Hippo/YAP pathways through Gαq or Gα12/13‐coupled receptor activation. Hippo pathway inhibition (by blocking Lats1/2 kinases)	Breast cancer Colon cancer	[111] [112]
Chemokine receptor (CXCR4)	CXCL12, SDF‐1	PI3K, ERK1/2, PLC, JNK Akt, Src, PIP2, IP3, Ras, Raf	HNSCC	[105]
Frizzled (Fz) PAR1 Parathyroid receptor1 (PTHR1)	Wnt 3A (canonical pathway) Thrombin or TFLLRN PTH	Stabilization of β‐catenin and its transcription activity by canonical Wnt signaling	Colon cancer Lung cancer Prostate cancer	[113, 114] [115, 116] [117]
Prostaglandin receptors (PE2, PE4)	PGE2	Cyclooxygenase pathway, PI3K (coupling to Gαi)	HNSCC Breast cancer Colon cancer	[118] [105] [119]
Lysophosphatidic acid receptors LPA1–6)	LPA	Rho‐dependent pathways β‐cantenin stabilization Kruppel‐like factor 5	Colon cancer Ovarian cancer Prostate cancer	[120] [121] [105]
Sphingosine 1‐1‐phosphate receptor (S1PR)	S1P	Ras‐ERK, PI3K/‐Akt/‐Rac, Rho, STAT3 (coupling to Gαi)	Glioma	[122, 123]
Angiotensin II type 1 receptor	Angiotensin II	TNF‐α, ERK1/2, NF‐κB, STAT	Gastric cancer Prostate cancer	[124] [105]
Bradykinin receptor Type 1 and 2 (B1R, B2R)	Kinins	Gαq and cross‐talk with EGFR Ras, Raf, ERK	HNSCC	[118]

*Abbreviations*: LPA: lysophosphatidic acid, PGE2: prostaglandin E2, CXCL12: C‐X‐C motif chemokine ligand 12 (also SDF‐1), ERK: extracellular signal‐regulated kinase, PI3K: phosphoinositide 3‐kinase, PLC: phospholipase C, PIP2: phosphatidylinositol 4,5‐bisphosphate, IP3: inositol 1,4,5‐trisphosphate, TNF: tumor necrosis factor, EGFR: epidermal growth factor receptor, NF‐Κb: nuclear factor kappa‐light‐chain‐enhancer of activated B cells, STAT: signal transducer and activator of transcription, JNK: c‐Jun N‐terminal Kinase, HNSCC: head and neck squamous cell carcinoma.

The intricate signaling networks that GPCRs mediate emphasize their importance as prospective therapeutic targets. It remains challenging to define GPCRs as cancer‐specific drivers due to the intricacy and variety of their expression and mutation patterns across numerous cancer types, despite the growing body of research on their role in cancer. On the other hand, hotspot mutations in G proteins have been shown to function as carcinogens in specific cancers. This review will examine the significance of G proteins and their role in cancer, highlighting their crucial role in disease development and potential as therapeutic targets.

### 5.1 Signaling Through Gαq G Proteins

The GNAQ oncogenes—a large number of the mitogenic signals acting on GPCRs—are triggered by growth factors and are transduced by heterotrimeric subunits of the Gαq family, which are encoded by GNA15, GNA14, GNAQ, and GNA11 and their associated receptors [105, 106]. Members of the Gαq family activate phospholipase Cβ (PLCB1‐4 4) and cleave PIP2 into diacylglycerol and IP3. By utilizing regulated kinases and transcriptional networks, the production of these second messengers triggers cytosolic calcium mobilization and initiates a range of downstream signaling cascades [107, 108, 109]. The original concept of mutating the Gαq subunit into a constitutively active, GTPase‐deficient type (Q209L and G207T) was the basis for earlier research investigating Ras oncogene mutations [110]. In addition to causing a malignant transformation in NIH3T3 cells, these Gαq mutants exhibited the ability to develop tumors in nude mice [110]. Recent attempts at cancer sequencing have indicated that activating mutations in Gα11 or Gαq are generally detected in more than 90% of uveal melanoma (UM), where they function as driving oncogenes, primarily at Q209. Since this initial finding, several clinical diseases, including Sturge‐Weber syndrome, congenital hemangiomas, and leptomeningeal melanocytic lesions, have been associated with abnormal Gαq activity [125, 126, 127, 128]. About 10% of cutaneous skin melanomas also include mutations in the Gαq family. Approximately 2500 persons in the US are affected by UM annually, making it the most common malignancy of the adult eye [128]. In UM, the oncogenic driver is the hyperactivation of the Gαq‐pathway, as almost 5% of UM patients without mutations in Gα11 or Gαq possess activating mutations in additional Gαq pathway levels that are cooperatively exclusive, for example, in Gαq‐coupled GPCR (CYSLTR2) or the downstream effector of Gαq PLCB4 [129, 130]. UM, resulting from CYSLTR2 mutation and hyperactivation, remains one of the most notable instances of a GPCR‐driven tumor type to date [131]. Investigations examining the mechanisms by which abnormal Gαq signaling contributes to carcinogenesis have shown that, in contrast to the transient activation of mitogenic kinases and second messengers following canonical Gαq stimulation, mutant Gαq needs an interaction between the protein‐Rho‐specific guanine nucleotide exchange factor (GEF) TRIO to maintain long‐term signaling [132]. TRIO phenocopy knockdown reduces Gαq expression in vivo in UM models based on their tumorigenic potential, stimulation of AP‐1‐mediated transcription, and activation of JNK and p38, as well as cell proliferation assays [132]. Notably, this signaling pathway activated a transcriptional coactivator, Yes‐associated protein (YAP), which is regulated by the Hippo pathway, without the assistance of PLCβ, the most well‐known Gαq target. It has been discovered that the Hippo pathway, which suppresses tumor growth and is widely involved in controlling organ growth and cell proliferation, is frequently altered in cancer [133]. It has been shown that YAP activity is essential for tumor development and proliferation in UM [134, 135]. According to recent research, focal adhesion kinase (FAK) regulates the activation of YAP, making it a crucial modulator of the oncogenic signaling pathway controlled by Gαq and a potential therapeutic target in UM105 patients. Like R183, Q209 is located in the GTP‐binding domain of Gαq, where mutations alter the inherent GTPase activity and enhance signaling activity. In contrast, compared with Q209 mutants, R183 mutants have a much lower expression potential. A class of other melanocytic neoplasms, including primary melanocytic tumors, Ota nevi, and blue nevi in the central nervous system, commonly contain activating mutations of Gαq. Moreover, they have also been connected to congenital hemangiomas [127, 136]. Numerous roles of Gαq in the immune system have been identified, including the potential to prevent tumor growth. In vitro survival and in vivo proliferation of B and T cells are improved by loss of GNAQ expression [137, 138, 139, 140]. Remarkably, recurrent loss‐of‐function (LOF) mutations at Y101or T96S or loss of Gαq expression affect the expression of the Gαq pathway in nearly 25% of natural killer (NK)/T/T cell lymphoma, an extremely malignant and highly aggressive subtype of non‐Hodgkin's lymphoma [140]. NK cells in mice specifically knocked out for Gαq showed an inherent survival advantage over WT NK cells, which is permanent, with the effects of Gαq deletion also observed in additional immune cells. Additionally, in Gαq‐low NK cell lines, WT Gαq expression induced apoptosis, which was inhibited by concurrent T96S Gαq expression, indicating that T96S Gαq functions as a dominantly negative mutant to enhance NK cell carcinogenicity [140]. The intricate molecular processes underlying Gαq‐driven carcinogenic signaling are highlighted by the propensity of Q209 and R183 Gαq hotspot mutations to occur in solid tumors, compared with the prevalence of Y101 and T96 in hematological cancers. This suggests a complex relationship between the tumor‐suppressive or carcinogenic functions of these mutations and the cellular environment in which they occur.

### 5.2 Signaling Through Gαs in Cancer

One of the most frequently mutated G proteins in human cancer is the GNAS oncogene, which codes for the Gαs protein [141]. Based on the description of mutations in several tumor types, GNAS occurs most frequently in appendix malignancies (70%) and pituitary tumors (27%), and it is mutated in around 5% of sequenced tumors [141]. Through TCGA, the most common mutations in GNAS are found in endometrial carcinomas (7.3%), colorectal malignancies (4.7%), adrenocortical carcinomas (5.5%), stomach adenocarcinomas (5.7%), esophageal carcinomas (4.9%), pancreatic adenocarcinomas (5.6%), and ulcerative carcinomas (5.7%). Most mutations in Gαs occur at a hotspot position (R201), resulting in the Gαs protein becoming constitutively active with impaired GTPase activity [142]. Q227 is a secondary hotspot; however, it is less common than R201 mutants. Studies investigating the Cholera toxin's mode of action were the first to propose the functional relevance of the R201 residue. It was discovered that the cholera toxin ADP‐ribosylates Gαs's R201, permanently blocking its GTPase activity, thereby activating its activation [143, 144]. Since then, Gαs crystallographic investigations have verified that the R201 residue is located in the GTP‐binding pocket and promotes GTP hydrolysis [145]. Adenylyl cyclases are stimulated when Gαs are activated, either by activating mutations or by stimulating Gαs‐coupled GPCRs. This results in the synthesis of cAMP, the activation of PKA, and additional signaling functions [146, 147]. Initially, several growth hormone‐secreting pituitary cancers with R201C/H mutations demonstrated the carcinogenic effects of Gαs [146, 148, 149]. Hormone‐insensitive growth resulted from sustained signaling triggered by mutant Gαs in these cancers, which bypassed the canonical necessity for growth hormone activation [142]. Indeed, around 7% of adrenocortical carcinomas in TCGA have a mutation in the Gαs subfamily (GNAS and GNAL). Surprisingly, a significant percentage of cancer types in TCGA exhibit extensive activation of the Gαs pathway, with gastrointestinal tract cancers showing a notable enrichment [150]. This is supported by the fact that 5.96% of stomach adenocarcinomas, 5.24% of colorectal cancers, and 5.03% of pancreatic cancers had mutations in the Gαs subfamily. Surprisingly, over 50% of patients with colorectal cancer exhibit abnormal Gs pathway activation, which includes downstream effectors of Gαs such as PKA and adenylyl cyclases, activating mutations or gene amplification of GNAS. Additionally, inflammatory prostaglandins (PGs) that accumulate locally due to COX2 overexpression lead to Gs‐coupled prostaglandin E2 (PGE2) receptors (PTGER2, 4) activation [151, 152]. Tumorigenesis and the development of colorectal cancer are thought to be facilitated by these genetic changes and autocrine activation, which are crucial in deregulating and activating the intestinal epithelium's PKA, MAPK, and Wnt pathways [153, 154, 155]. It is interesting to note that tumors with Gαs mutations frequently have a very mucinous appearance. This is consistent with research showing that mucin genes are upregulated in response to GNAS expression [156]. Among gastrointestinal mucinous neoplasms, GNAS mutations are prevalent in intraductal papillary mucinous neoplasms, a type of invasive pancreatic cystic neoplasm that has the potential to progress into appendiceal mucinous tumors or pancreatic adenocarcinoma, where Gαs‐promoted signaling is essential for tumor growth [156, 157, 158, 159]. Gαs and their downstream signaling pathways are crucial regulators within stem cell populations, where they prevent self‐renewal and play a role in the development of mucinous neoplasms. Notably, Gαs can have different functional effects depending on the cellular environment. Gαs can also function as a tumor suppressor in some stem‐like cell states; however, gain‐of‐function mutations in Gαs usually have pro‐oncogenic effects. The complex role of Gαs in cancer biology is further highlighted by the reality that LOF mutations or deletions of Gαs may expedite tumor initiation and progression by de‐repressing critical oncogenic pathways, including the Hippo signaling pathway and Sonic Hh [160, 161].

### 5.3 Gαi/o Protein‐Encoding Gene Mutations in Cancer

Activation of PI3K and MAPK signaling is among the many effectors through which the G proteins’ Gαi/o subfamily initiates signaling. Gαi inhibits adenylyl cyclase, which lowers cAMP in contrast to Gαs signaling [162]. Activation of the islet‐activating protein (IAP), also known as pertussis toxin, can indeed inhibit adenylyl cyclase activity, resulting in a reduction in cAMP levels. Treatment with IAP reversed this inhibition, leading to an increase in cAMP production. Adenylyl cyclase activity was increased, and Gαi was rendered inactive by IAP ADP‐ribosylation, as reported by various groups that studied the IAP substrate [163, 164, 165]. Constitutively active Gαi mutants have been proven to have the capacity to change cells; therefore, they are recognized as proto‐oncogenes, just like other G proteins [166, 167]. It has also been discovered that when expressed in cells, activated mutations originating from cancer of GNAO1, which codes for Gαo, induce anchorage‐dependent growth and oncogenic transformation [168]. Hotspot‐activating mutations in GNAI2, also known as the gip2 oncogene, have been identified in a variety of cancer types, encompassing adrenal and ovarian tumors, and have been demonstrated to cause dysfunction in a subgroup of growth hormone‐releasing pituitary adenomas. Nevertheless, because there are so few patients with this specific disorder, little research has been conducted on the impact of germline GNAI variants, which warrants additional investigation [169, 170]. Accordingly, it has been discovered that breast‐invasive cancer has an elevated level of GNAI2 (encoding Gαi2) [141]. The most frequently harmfully mutated class of GPCRs across malignancies is Gαi/o‐coupled receptors [150]. Furthermore, it has been found that Gαs‐activating mutations and Gαi/o‐coupled receptor inactivating mutations are mutually exclusive. This implies that they produce identical functional results, convergent on elevated cAMP activity [150]. The functional significance of these alterations has not yet been thoroughly investigated, despite a significant percentage of GPCR mutations in GI‐tract cancers being Gαi‐linked. The Gαi/o subfamily of G proteins is altered at indistinguishable frequencies in GI cancers as those of the Gαs subfamily [141]. In GI malignancies, corresponding to the frequent occurrence of Gαs‐pathway activation, elevated cAMP/PKA activity may be a common mechanism that causes tumors in these tissues and warrants further research from both signaling and therapeutic perspectives.

### 5.4 Gα12 Protein‐Encoding Mutations in GNA13 and GNA12 in Cancer

GNA12 and GNA13 encode the two α subunits that comprise the Gα12 subfamily of G proteins. Expression in nearly every tissue type, Gα12 and Gα13 are necessary for cytoskeletal remodeling, including cell proliferation, adhesion, polarity, migration, and as well as invasion [171, 172]. After research showed that Gα13 had vigorous transforming activity, it identified GNA13 and GNA12 as active mutants, collectively known as the gap oncogene [173, 174]. Since then, Gα12/13 have been extensively investigated as a factor driving cellular transformation, cancer development, and metastasis in various cell types [175, 176, 177]. Leukemia‐associated RhoGEF, PDZRhoGEF, and p115RhoGEF are among the members of the RhoGEF family that mediate signaling to Rho in response to Gα12/13 [178, 179, 180, 181]. Early research on how an active mutant of Gα12 fully transformed fibroblasts showed that Gα12 extensively promoted transcriptional activity through the serum response element and the c‐fos promoter element, dependent on Rho [182]. According to additional research, Gα12/13 can signal through several effectors, including MAPK, Radixin, and β‐catenin [171, 183]. Collectively, these signaling pathways regulate numerous transcriptional networks and cancer‐related cellular functions, including the activation of AP‐1, STAT3, and YAP [183, 184, 185]. Overexpression or mutation of Gα13/13 or Gα12/13‐linked GPCRs, such as thromboxane A2 receptor TA2R (TBXA2R) or protease‐activated receptor 1 (PAR1) (F2R), can induce aberrant Gα12/13‐driven signaling, which is transformative and dramatically increases the possibility for invasion of several cancer types [175, 186, 187, 188]. The elevated expression of Gα12/13 and Gα12/13‐coupled GPCRs, along with their effectors, such as Rho, is significant in various cancer types, including hepatocellular, prostate, and breast cancers [189, 190]. It has been discovered that the expression levels of GNA13, specifically elevated in several solid tumors, influence therapeutic resistance in squamous cell malignancies, including head and neck squamous cell carcinoma [191, 192, 193]. It is well known that many chemokine receptors, such as CXCR4, and receptors for bioactive lipids, such as LPAR and S1PR (S1PR2–5), can link to Gα12/13 and contribute to cancer's metastatic potential [193, 194]. Remarkably, it has been discovered that the Gα13/RhoA signaling axis has a tumor‐suppressive function in various hematological and lymphoid cancers, including diffuse large B cell lymphoma and Burkitt's lymphoma [195, 196]. Although it has not been adequately analyzed and identified as such by OncoKB188, GNA13 seems to have a possible hotspot mutation at R200 throughout TCGA PanCancer investigations. Only bladder urothelial carcinomas include this cluster of mutations, though their function remains unidentified [150]. Specifically, GNA13 is a significantly mutated gene in B‐cell lymphomas originating from germinal centers, which is prompted by a subpopulation of B lymphocytes with abnormal growth and dissemination that are firmly confined to germinal centers in lymphoid tissues and are highly regulated [197, 198]. Although the accurate processes by which the Gα13 signaling pathway's inactivation permits the formation of lymphomas are still unknown, numerous studies have shown that the reduction of the Gα13/RhoA axis causes B cells to produce higher amounts of phosphorylated AKT [197].

### 5.5 Mutations that Impact RGS and Gβγ in Cancer

Functional elements of G protein signaling, such as the βγ subunits of heterotrimeric G proteins and members of the RGS family, can also enhance or alter G protein‐driven signaling, leading to oncogenesis [199]. Due to their inherent GAP activity, members of the RGS family physiologically inhibit G proteins. They facilitate the rearranging and inactivation of the heterotrimeric G protein complex 5 and increase GTP hydrolysis by the Gα‐subunit. Numerous RGS protein mutations and transcriptomic dysregulation, particularly those resulting in LOF, have been identified in recent pan‐cancer analyses. This has promoted G protein activity through a mechanism of G protein signaling potentiation and a previously unknown tumor suppressive role [200, 201]. Numerous cancers, particularly hematological malignancies, have been found to harbor recurrent mutations in Gβ proteins, including GNB1 and GNB2, which lead to abnormal stimulation of the MAPK and PI3K/AKT pathways [202]. The impact of specific Gβ mutations may be determined by lineage‐dependent cellular predispositions, as demonstrated by the evident, unique mutations that group cancer types according to cell lineage. It has also been discovered that Gβγ subunits promote cell migration and metastasis. PREX1, a GEF that stimulates the small GTPase RAC, can be directly bound by Gβγ and cooperatively activated with PIP3 [203, 204]. Interestingly, Gβγ expression is required for PI3Kβ's transforming ability; p110 mutants with binding defects have been shown to significantly decrease cells' ability to transform and exhibit GPCR‐driven chemotaxis [205, 206]. This mutation dramatically reduced extravasation, matrix degradation, and macrophage‐stimulated invasion of tumor cells in breast cancer cells, which is consistent with these findings and suggests that Gβγ may play a potential function in paracrine communication among immune cells and tumor cells [207, 208]. In this regard, a growing body of research indicates that Gβγ signaling plays a crucial role in cancer metastasis and carcinogenesis, partly by promoting and modifying a prometastatic TME [209, 210].

In summary, mutations in GPCRs and their associated G proteins contribute significantly to cancer by driving constitutive signaling and promoting tumor growth, survival, and metastasis. Hotspot mutations in genes such as GNAS, GNAQ, and GNA13 underscore the oncogenic potential of aberrant GPCR signaling. Understanding these alterations offers promising avenues for targeted cancer therapies.

## 6 Cancer‐Associated GPCR‐Mediated Signaling Pathways

In both primary and metastatic tumor cells, various GPCR‐mediated signaling pathways are involved, linking their activation by circulating or locally generated ligands to the development of cancer. A brief description of some of these signaling pathways is given below.

### 6.1 Wnt Signaling

The signaling pathway and tissue homeostasis are mediated by the Fz receptor, also referred to as the Wnt receptor. Wnt proteins play a crucial role in both physiological development and malignant processes, such as cancer. By antagonizing the β‐catenin “destruction complex,” which is made up of adenomatosis polyposis coli (APC), E3 ubiquitin ligase part TrCP1, glycogen synthase kinase 3, Axin, and casein kinase 1, through the Fz‐lipoprotein‐related protein 5/6 (LRP5/6) receptor complex, the canonical Wnt signaling pathway stabilizes β‐catenin. In the absence of Wnt, the “destruction complex” persistently breaks down β‐catenin, the main effector of this pathway. After stabilizing, β‐catenin is transported to the nucleus of the cell, where it contributes to controlling proliferation and differentiation. LPA receptors (LPA1–6), PG receptors, PTHR1, and endothelin receptors (ET1‐4) are additional GPCRs actively participating in the β‐catenin stabilization pathway.

**FIGURE 4:**
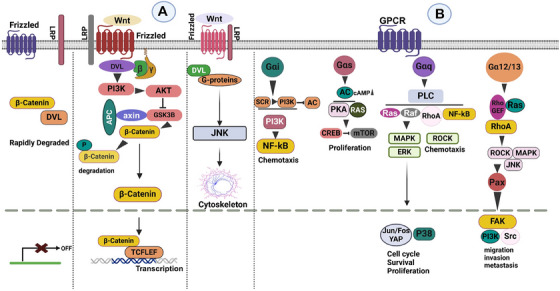
Wnt/β‐catenin pathway and dysregulation of GPCR signaling in cancer. (A) The Wnt/β‐catenin pathway is characteristic of the Frizzled (FZ) class F GPCRs, stimulated by Wnt (Wingless/Int1) proteins and activating G protein‐independent (DVL) and G protein‐dependent pathways. The canonical Wnt pathway activates β‐catenin signaling by binding Wnt to the Fz and LRP5/6 receptors. Without Wnt, β‐catenin is degraded by the “degradation complex.” Wnt binding disrupts this complex, stabilizing β‐catenin, facilitating its nuclear translocation, and promoting transcriptional activation via Lef/Tcf factors. Noncanonical Wnt signaling (e.g., Wnt5a) is mediated via Fz, affecting, among others, the activation of JNK and the cytoskeleton. (B) GPCR dysregulation in cancer is illustrated, showing its contribution to cancer hallmarks, including uncontrolled proliferation, evasion of apoptosis, and metastasis. *Abbreviations*: GSK3β—glycogen synthase kinase 3 beta, TCF/LEF—T‐cell factor/lymphoid enhancer‐binding factor, JNK—c‐Jun N‐terminal kinase, NF‐κB—nuclear factor kappa B, mTOR—mechanistic target of rapamycin, MAPK—mitogen‐activated protein kinase, ERK—extracellular signal‐regulated kinase, ROCK—Rho‐associated coiled‐coil containing protein kinase, RhoA—Ras homolog family member A, FAK—focal adhesion kinase, Src—proto‐oncogene tyrosine‐protein kinase Src, YAP—Yes‐associated protein, P38—p38 mitogen‐activated protein kinase.

Due to either oncogenic alterations in its phosphorylation site at the N‐terminus or the mutational ablation of its negative regulators, Axin or APC, hyperactive stabilized β‐catenin is present in many cancers [211, 212]. Particularly in colon, breast, lung, oral, cervical, and hematological cancers, activated β‐catenin may be carcinogenic. Furthermore, Wnt signaling promotes carcinogenesis by affecting the TME through subtle interactions between invasive immune cells, such as leukocytes, and altered cells. Additionally, Wnt signaling contributes to the maintenance of cancer stem cells through the epithelial–mesenchymal transition (EMT). Although it is also transduced by Fz receptors, noncanonical Wnt signaling does not need β‐catenin/Tcf activity or the LRP5/6 coreceptor. Prototypes of this Wnt pathway include Wnt5A and Wnt5B [213]. Cancer, planar cell polarity, tissue regeneration, dorsoventral patterning, and convergent extension movements are among the developmental processes in vertebrates that are impacted by noncanonical Wnt signaling. Noncanonical signaling generally opposes canonical Wnt/β‐catenin signaling and facilitates the Rho‐associated protein kinase (ROCK) pathway, a key regulator of the cytoskeleton. The Wnt–Ca2+ pathway, which controls TAK1‐induced Nemo‐like Kinase and nuclear factor of activated T cells and is involved in cancer development, is another illustration of β‐catenin‐independent signaling. Stromal cells secreted growth factors within the TME, which impact Wnt/β‐catenin signaling. In colorectal cancer cells, for example, stimulation of hepatocyte growth factor has been shown to facilitate tyrosine‐based β‐catenin phosphorylation and its dissociation from Met through the PI3K pathway, promoting tumor development and invasion [214]. Additionally, PDGF, TGF‐β, and EGF stimulated β‐catenin translocation, leading to EMT [215]. In mouse models, vascular endothelial growth factor (VEGF)‐dependent angiogenesis was enhanced by Wnt/β‐catenin signaling [216]. The accurate role of G proteins remains an intriguing and unresolved issue in Fz‐mediated Wnt/β‐catenin signaling. Despite some studies suggesting that Wnt signaling is affected by G proteins [217, 218, 219], other studies have failed to demonstrate that G proteins are an essential component of Wnt/β‐catenin signaling [220, 221]. Research using transfection of MEF cells showed that Gαi and the Wnt/β‐catenin pathway did not interact [221]. G proteins have various effects on modulating Wnt/β‐catenin signaling; however, the study showed that they are insufficient to increase Wnt/β‐catenin signaling in MEF cells when exogenous Wnt3a is present. Gαs enhances Wnt/β‐catenin signaling, decreased by Gαq and Gα13, and unaffected by Gαi in the same experimental setup. G proteins are likely necessary for the Wnt/β‐catenin signaling pathway in all cell types, given their importance as key components. On the other hand, the scientists concluded that Gα proteins are not essential for pathway transduction and are not a component of the core Wnt/β‐catenin signaling pathway [221]. Consequently, the function of G proteins in Wnt signaling pathways remains a contested topic (Figure 4A).

### 6.2 Hippo Signaling Pathway

The oncoprotein is the transcriptional coactivator with a PDZ‐binding motif (TAZ)/Hippo– YAP pathway. It has become an essential conserved system that controls polarity, cell adhesion, mechanical and cytoskeletal proteins, organ size, and cell growth and transformation [222]. Cancer develops as a result of this mechanism being dysregulated. Two crucial downstream effectors of Hippo signaling that play a significant role in cancer are the protein YAP and its homolog, TAZ. Researchers are developing pharmacological inhibitors of TAZ and YAP, two key targets for cancer therapeutics. The oncogenic YAP pathway is triggered when the tumor‐suppressing Hippo pathway is disrupted, which limits the nuclear localization and transcriptional activity of YAP/TAZ. Once the Hippo enzymatic cascade is blocked, YAP/TAZ are released from their cytoplasmic anchoring point and migrate to the cell nucleus. They function as transcription coactivators in the nucleus by binding to transcription factors of the TEAD family, which activates downstream target genes and promotes oncogenicity. The Mst1–2–Lats1/2 kinase cascade of the Hippo pathway directly phosphorylates YAP/TAZ, which causes cytoplasmic retention through 14‐3‐3 binding. This encourages TrCP‐mediated ubiquitination and destruction of YAP/TAZ. Following the initial finding of LPA–YAP/TAZ and sphingosine 1‐phosphate (S1P) activity, GPCRs were identified as efficient YAP oncogenic pathway inducers in the pursuit of physiological YAP/TAZ [133, 223, 224]. It has been demonstrated that GPCRs linked to cell proliferation increase the transcriptional activity of the coactivator YAP [133, 135, 222, 225]. There are several methods in which GPCRs have been shown to control the Hippo pathway. Through the Gα12/13, Gαq, or Gαi routes, LPA and thrombin receptors lead to the activation of YAP/TAZ. However, epinephrine and glucagon receptors mediate the Gαs pathway, which inhibits the YAP/TAZ pathway. Through Gα12/13, It has been demonstrated that GPCRs reduce LATS activity. Consequently, YAP is released from LATS‐dependent suppression [133]. Based on the Gutkind group's study, activation of YAP via oncogenic mutations in Gαq through actin polymerization and a mechano‐sensing pathway instead of interfering with the Hippo‐suppressing pathway [123]. PKA mediates upstream signals by preventing the synthesis of actin fibers or by directly phosphorylating LATS1/2 [226, 227]. PKC seems to have particular effects on its isoforms; for instance, while novel PKC isoforms reduce YAP/TAZ activity, classical PKC isoforms increase it [228]. While MST1/2 does not appear to be a direct target of GPCR signaling, MAP4Ks‐mediated LATS1/2 phosphorylation responds to a range of GPCR ligands [229, 230]. Consequently, G protein and GPCR activity in Hippo signaling is most likely tissue‐dependently influenced by Rho GTPases, actin cytoskeleton remodeling, and protein kinases (including PKA and PKC). Nevertheless, the mechanism is still unknown [231]. Furthermore, according to current research, YAP/TAZ have been identified as the downstream effectors in the noncanonical Wnt signaling pathway, which includes Wnt–FZ/ROR, G12/13‐Rho GTPases, and Lats1/2. This pathway promotes TEAD‐mediated gene transcription and the carcinogenic effects of YAP/TAZ.[232]. Additionally, Hh ligands diminish YAP/TAZ through the SMO–Gαs–cAMP–PKA signaling pathway [160]. These results suggest that atypical GPCRs (FZ, SMO) regulate the Hippo pathway and the interactions between the Hippo pathway and other critical pathways in cancer progression. Moreover, GPCRs’ impact on the activity of YAP/TAZ, regulated by PKD and PI3K downstream of the insulin receptor, is enhanced in the presence of insulin [233]. It has also been shown that MAPK signaling modulates the Hippo pathway [234]. Given that YAP/TAZ‐induced transcriptional activities are heavily involved in cancer, weakening YAP and TAZ can be a feasible treatment and prevention strategy for a variety of cancers. A possible approach might be to lower the YAP dosage by using shRNA depletion. When the shRNA‐induced mortality of a large panel of human cancer cell lines was investigated, it was demonstrated that cancer cell lines with WNT signaling stimulation are especially susceptible to YAP knockdown [235]. Due to this, YAP suppression does not always correspond with its conduct, and blocking YAP may involve critical TEAD‐independent interactions that are particular to some tumor cells.

**FIGURE 5:**
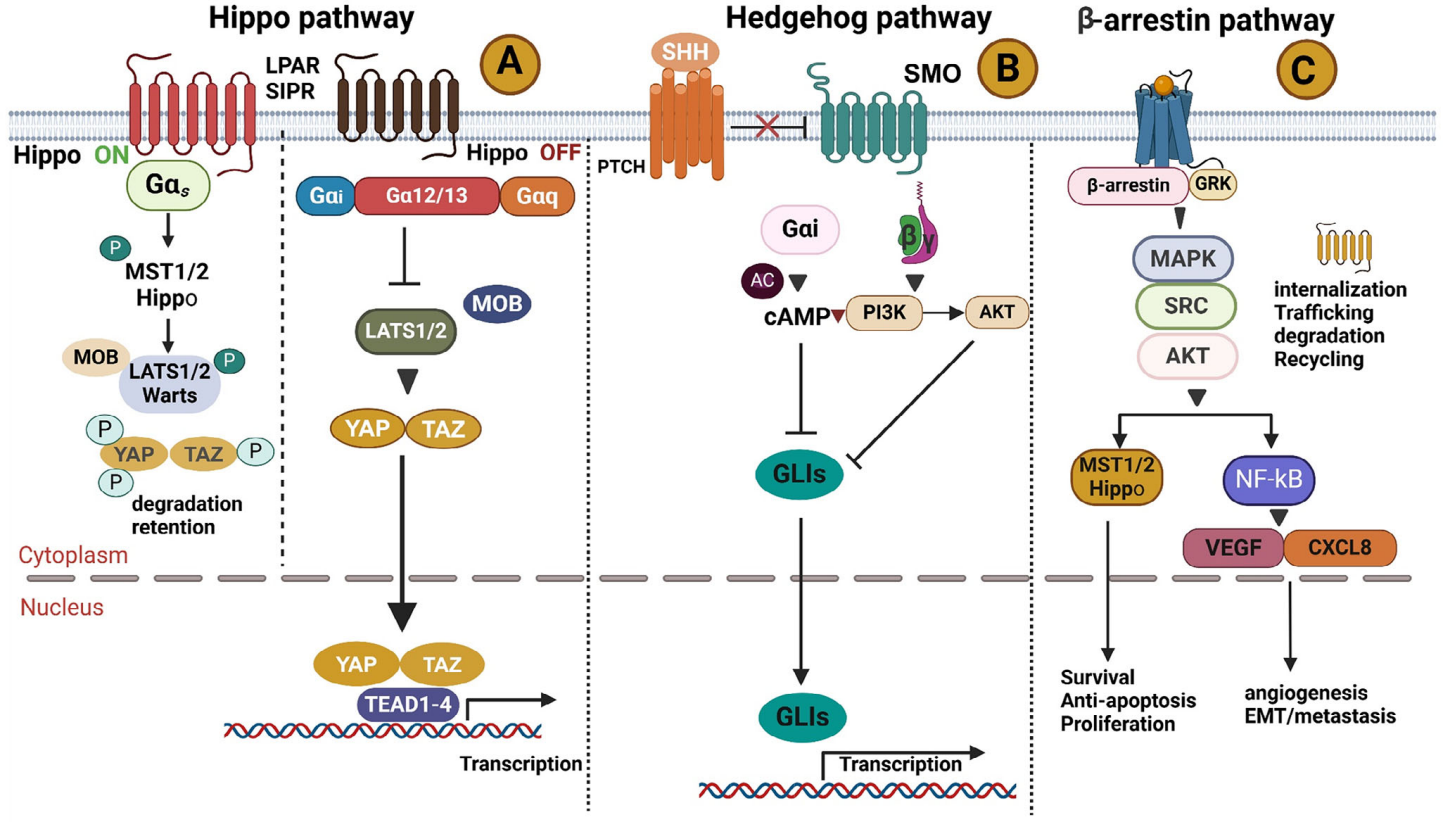
Key GPCR‐associated pathways in tumorigenesis. (A) Hippo signaling pathway: LATS1/2 kinases phosphorylate YAP/TAZ, thereby inactivating them and restricting their nuclear entry. Gαs activates Hippo (inhibiting YAP/TAZ), while Gα12/13, Gαi, and Gαq/11 activate YAP/TAZ, promoting tumorigenesis. (B) Hedgehog pathway: In the absence of Hh, PTCH inhibits SMO, thereby blocking GLI activation. When Hh binds to PTCH, SMO is activated, leading to the activation of Gαi signaling and, in turn, activating GLI‐driven transcription that promotes tumor growth. (C) β‐arrestin‐mediated signaling: β‐arrestins (β‐arr1, β‐arr2) modulate GPCR desensitization by inhibiting G proteins while activating G protein‐independent pathways. These include ERK1/2, PI3K/AKT, and Hedgehog signaling, which are critical for cancer progression. This panel highlights the dual role of β‐arrestins in regulating GPCR signaling and promoting tumor growth and metastasis. *Abbreviations*: MST1/2—mammalian sterile 20‐like kinase 1 and 2, LATS1/2—large tumor suppressor kinase 1 and 2, YAP—Yes‐associated protein, TAZ—transcriptional coactivator with PDZ‐binding motif, TEAD1—TEA domain transcription factor 1, SHh—sonic hedgehog, PTCH—patched receptor, GLI—glioma‐associated oncogene homolog, VEGF—vascular endothelial growth factor, CXCL8—C‐X‐C motif chemokine ligand 8.

Additionally, medications that target GPCRs and G proteins may reduce YAP/TAZ activation and decrease the spread of cancer because the Hippo pathway is highly mediated by GPCR‐regulated downstream signaling. Gαs‐targeted substances, for instance, may similarly reduce YAP/TAZ function to epinephrine, dobutamine, and glucagon [236, 237]. Antagonizing or decreasing Gα12/13‐, Gαq/11‐, or Gαi/o‐mediated signals, as well as employing monoclonal antibodies (mAbs) specific for LPA or S1P and phosphatase‐resistant LPA analogs [238, 239], may decrease the YAP/TAZ function. It has recently been demonstrated that the cyclic depsipeptide FR900359 binds to mutant Gαq and inhibits the downstream effectors of MAPK and YAP [240, 241]. Moreover, it has been reported that phosphodiesterase inhibitors such as Rolipram or forskolin activate PKA and inhibit YAP/TAZ [160, 238]. Also, PKC inhibitors can reduce YAP/TAZ activity depending on the type of cell [242]. However, it has been demonstrated that several GPCR‐based drugs, such as dopamine and blockers, have significant effects on cardiac and psychological functions; as a result, adverse effects must be addressed before using these drugs in cancer treatment [243, 244] (Figure 5A).

### 6.3 The Hh Pathway

The Hh pathway is essential in stem cell maintenance and embryonic development, and is activated in tumors, and its deregulation may lead to neoplastic transformation [245]. The GPCR SMO, the tumor suppressor patched (PTCH) cell surface receptor, and the transcription factors for glioma‐associated oncogenes (GLI) are the primary elements of this cascade. PTCH inhibits SMO activity when it is quiescent (unstimulated), which prevents GLIs from activating and becoming ubiquitinated. To promote proliferation and survival during carcinogenesis, Hh releases SMO upon binding to PTCH, which stimulates Gαi signaling and activates GLIs. The GPCR PTCH and SMO are important targets for regulation, as inactivating PTCH mutations or activating SMO mutations are linked with medulloblastoma and basal cell carcinoma [246]. Despite mounting evidence of an SMO‐independent variant Hh route, SMO inhibitors have evolved to treat basal cell carcinoma. To address this, SMO/GLI multitarget antagonism is a practical approach to targeting medulloblastoma [247, 248] (Figure 5B).

### 6.4 GPCR–β‐Arrestin pathway

β‐Arrestins have emerged as key players in cancer‐related GPCR signaling, extending their traditional role in receptor desensitization. In various tumor types, they influence critical cellular behaviors such as cytoskeletal reorganization, migration, and transcriptional regulation. Their context‐ and isoform‐specific roles, particularly those of β‐arrestin 1 and β‐arrestin 2, vary across different cancer types and are influenced by the TME. Figure 5C illustrates how β‐arrestins interact with diverse intracellular effectors and signaling hubs, contributing to both tumor‐promoting and suppressive outcomes. Their involvement in processes such as metastasis, immune evasion, and nuclear signaling highlights their complexity in cancer biology. Continued investigation into β‐arrestin functions may help identify novel intervention points that are distinct from traditional GPCR–G protein signaling pathways.

### 6.5 Mitogen‐Activated Protein Kinases

MAPK signaling is essential for proliferation and survival. The proteins Ras and Raf, known to contain distinctive oncogenic alterations, are downstream of the layers of kinases that make up the MAPK pathways [249]. This pathway is linked to tumor‐promoting activity and is activated by the Gαq, Gαi, and Gβγ subunits that are regulated by GPCRs (Figure 4B).

### 6.6 PI3K–AKT–mTOR/NF‐κB

Cell migration, survival, growth, and metabolism are all influenced by the PI3K–AKT–mammalian target of rapamycin (mTOR)/NF‐κB pathway. It frequently exhibits dysregulation throughout tumor development, supporting proliferative signaling, preventing cell death, and facilitating immune evasion. The PI3K/Akt/NF‐κB axis can be activated by GPCR signaling through Gαq and Gβγ upon activation. This pathway enhances the expression of interleukin‐8 (CXCL8) and VEGF, which promotes key cancer hallmarks, including angiogenesis and metastasis. The EMT, a crucial step in metastasis, is mediated by the PI3K/Akt signaling pathway. For example, GPR120 stimulates the development of breast cancer [250]. These oncogenic signaling events are triggered by GPR137 (orphan, constitutively active), which causes ovarian cancer [251]. Alternatively, Gαi can activate the PI3K pathway through the chemokine receptor CXCR4, leading to NF‐κB activation and chemotaxis [252]. The serine/threonine kinase mTOR, which regulates cell growth, survival, and metabolism, particularly in cancer cells, can be activated by the protein kinase AKT. This cascade also receives signals that lead to the suppression of apoptosis. Gαs‐coupled GPCRs phosphorylate Raptor, a mTOR‐associated protein component of the mTOR complex (mTORC1), via PKA, which inhibits mTOR [253]. Therefore, one strategy to block the mTOR complex and reduce its proliferative function in cancer may be to activate Gαs‐coupled GPCRs (Figure 4B).

### 6.7 RhoGEFs (guanine exchange factors)

RhoGEFs are signaling mediators triggered by Gα12/13‐coupled GPCRs. By promoting tumor growth, angiogenesis, and metastasis, they activate key transcription factors associated with cancer. The Cholecystokinin B receptor (CCKBR) in pancreatic cancer signals through Gα12/13 when its ligand, Gastrin, activates it. Through the phosphorylation of FAK, the downstream effector ROCK of Gα12/13 is activated, leading to the phosphorylation of paxillin, a protein involved in cell adhesion and migration. Paxillin contributes to the EMT and proliferation, whereas FAK modulates downstream elements, including PI3K and Src [254]. aGPCRs have been shown to activate Gα12/13 proteins and are involved in cell adhesion to the extracellular matrix (ECM) and cell‐to‐cell communication. Among other malignancies, mutations and altered expression of aGPCRs have been identified in the prostate, colon, lung, and breast [255] (Figure 4B).

In summary, diverse GPCR‐mediated signaling pathways, such as Wnt, Hh, PI3K–AKT–mTOR, MAPK, and β‐arrestin pathways, play critical roles in tumorigenesis by regulating proliferation, survival, invasion, and immune modulation. The complexity and redundancy of these pathways underscore the challenges and opportunities in targeting GPCRs for cancer therapy. A deeper understanding of the pathway‐specific roles of GPCRs may guide the development of more effective, targeted interventions with reduced off‐target effects.

## 7 GPCRs in the Hallmarks of Cancer

GPCRs play essential roles in various physiological processes, and their dysregulation is increasingly recognized as a key driver in cancer. They contribute to major hallmarks of cancer, including uncontrolled proliferation, resistance to apoptosis, invasion, metastasis, angiogenesis, and immune evasion. Upon activation by ligands such as chemokines, hormones, and bioactive lipids, GPCRs trigger complex signaling cascades that reshape the TME and influence tumor–host interactions. This section focuses on their roles in migration, invasion, angiogenesis, and immune regulation.

### 7.1 Role in Migration, Invasion, and Metastasis

One of the most significant challenges in cancer treatment is metastasis, the process by which tumor cells migrate to other organs through the bloodstream or lymphatic vessels [256]. Instead of spreading randomly, tumor cells can selectively metastasize to particular organs with a higher prevalence [257]. By altering the cytoskeletal dynamics and structure of cells bearing receptors, chemokines are known to direct cell migration and facilitate metastasis. Furthermore, locally produced chemokines within the TME recruit leukocytes and macrophages, thereby increasing the cytokine‐rich environment and reducing the secretion of matrix metalloproteases (MMPs), which in turn promote the invasion, survival, and proliferation of cancer cells. Furthermore, organ‐specific metastasis is strongly correlated with GPCRs coupled to chemokine receptors in various types of cancer. Research has demonstrated that cancer cells with altered expression of chemokine GPCRs exploit chemokine migration, facilitating metastasis to multiple organs [258]. Among the most well‐known receptors for chemokines, CXCR4 is involved in migration, survival, and proliferation. It is frequently overexpressed in many types of cancer, which aids in metastasis. The widespread metastatic sites, like the bone marrow, liver, lungs, and lymph nodes, demonstrate that CXCL12/SDF‐1 is the chemokine ligand for CXCR4.[257]. It was shown that breast cancer cells express CXCR4 abundantly. Rac1 is activated by its activation through P‐REX1, which contributes to the spread of most breast‐type malignancies. Additionally, CXCR4 may link to G12/13, promoting metastasis in basal‐like breast cancer cells in a RhoA‐dependent manner [194]. Targeting the downstream signaling or the molecule regulating CXCR4 expression on tumor cells could provide therapeutic alternatives. It has been demonstrated that additional chemokine receptors, including CCR7 and CCR10, directly contribute to the proliferation, survival, and metastatic homing of cancer cells [259]. Additionally, several new investigations are underway to better understand the adhesion family of GPCRs and their potential contributions to the development and spread of cancer [260]. According to recent research, GPR116, a member of the mainly unidentified aGPCR family, activates the Gαq‐RhoA‐Rac1 pathway, contributing to the invasion and migration of breast cancer cells.

### 7.2 GPCRs in Tumor‐Induced Angiogenesis

Nag et al. [261] recently reviewed the different aspects of the cardinal GPCRs associated with tumor angiogenesis. Tumors release angiogenic substances that promote the migration and proliferation of endothelial cells, thereby forming new capillaries to meet the increased oxygen and nutritional needs of tumor cells. Numerous angiogenic substances, including S1P, PGs, thrombin, and chemokines, act on GPCRs expressed in endothelial cells [262, 263, 264]. Specific chemokines, including CCL5, CXCL8/IL‐8, and CCL2, can attract macrophages and leukocytes to the cancer site, where they might produce other angiogenic factors and VEGF that promote the formation of new blood vessels [263]. Moreover, the TME's inflammatory cytokines increase the production of COX‐2 and the local release of PGE2, which in turn increases the expression of proangiogenic VEGF, CXCL8, and CXCL5 in tumor and stromal cells [265]. By increasing the proliferation of endothelial cells, GPCRs and their ligands can directly stimulate angiogenesis. Alternatively, they can indirectly stimulate the production of other angiogenic factors and VEGF from immune, malignant, or stromal cells. Tumor vascularization enables pathways for invasion, metastasis, and the delivery of nutrients necessary for tumor growth.

### 7.3 TME Remodeling to Promote Immune Escape

The TME is a unique niche inhabited by a variety of cell types, including stromal cells, immune cells, cancer cells,  ECM, and diverse secreted substances, such as exosomes and microRNAs [266, 267]. Tumor growth, metastasis, and treatment outcomes are correlated with the altered TME landscape [268]. The TME score, an advanced TME infiltration pattern of GC, was recently established by evaluating cancer‐associated fibroblasts (CAFs) and 22 immune cell types, which are connected to pathologic and genomic features [266]. CAF biology and function have become a field of continuous, active research, and have been previously examined [269, 270]. The chemokines and their receptors are the most significant methods involved, and the compositions of infiltrating immune cells within the TME differed widely [271]. Interestingly, tumor, immunological, and stromal cells can all express chemokines and chemokine receptors [272]. The composition of TME immune cells was influenced by changes in chemokines and their receptors. To evade immune surveillance and eradication, they altered the immune responses, some of which were taken over by tumor cells [273]. The induction of immune cells, primarily dendritic cells (DCs), CD8+ T cells, NK cells, and M1 macrophages, triggered antitumor immune responses. GCs with elevated CXCR3 expression showed enhanced DC and T cell infiltration. The recruitment of DCs depends on the CXCR3/CXCL4 or CXCR3/CXCL4L1 axis, as these cells strongly stimulate T cells and activate the related humoral response, resulting in antitumor effects [274, 275]. Likewise, CXCR3 is essential for CD8+ T cell infiltration, which causes direct damage to tumor cells once the cells differentiate into cytotoxic CD8+ T cells [276, 277]. Furthermore, professional killer cells, or NK cells, are accumulated in the TME as a result of increased CXCL10 and CXCL12 signaling via CCR7 or CXCR3 [278, 279].

In contrast, chemokine signaling also plays a role in forming an immunosuppressive TME, allowing tumors to evade detection. This mechanism is primarily associated with the infiltration of several protumor immune cell types, including granulocytic (or PMN‐) myeloid‐derived suppressor cells (MDSCs), M2 macrophages, regulatory T (Treg) cells, and monocytic myeloid‐MDSCs [271, 280]. CCL22, produced mainly by tumor cells (or macrophage‐mediated), interacts with the receptor CCR4 on the surface of Treg cells to increase their number in the TME. Treg cells promote migration in response to CCL28 by another receptor, CCR10 [281, 282]. Furthermore, recruited monocytes can produce nonpolarized macrophages (M0) that can differentiate into two main subtypes: M1 and M2 macrophages. These two subtypes play very diverse roles in cancer. A variety of chemokine signals influences these transitions. Tumor‐derived chemokine releases, including CCL2, CCL3, CCL4, CCL5, CCL20, and CCL18, were necessary for active monocyte recruitment. Furthermore, blocking the CCL2‐CCR2 circuit resulted in an increase in M2 macrophages, while CCL11 favored macrophages toward an M2 phenotype [283, 284]. Other GPCRs also mediate the regulation of immunological responses. For example, through their respective GPCRs, EP1‐EP4 (PTGER1‐4), PGs can promote inflammation. The cyclooxygenases COX‐1 and COX‐2 produce PGs, especially PGE2. Nonsteroidal anti‐inflammatory medicines are examples of enzyme inhibitors used to reduce the incidence of some cancers and relieve pain. PGE2's ability to trigger inflammation by activating additional signaling pathways, including the Wnt, EGFR, and Toll‐like receptor/MyD88 pathway, has been thoroughly investigated [285, 286].

GPCRs play multifaceted roles in driving key hallmarks of cancer, including sustained proliferation, invasion, metastasis, and angiogenesis. Through interactions with chemokines, adhesion molecules, and diverse signaling pathways, GPCRs modulate both tumor cell behavior and the TME. Their ability to orchestrate organ‐specific metastasis and influence immune cell infiltration highlights their clinical relevance. Understanding these mechanisms offers promising therapeutic opportunities to disrupt cancer progression at multiple levels.

## 8 Recent Advances in Therapeutic Development Targeting GPCR Signaling Bias

Recent advances in therapeutic development targeting GPCR signaling bias have revolutionized the field by enabling more precise modulation of receptor pathways. Rather than uniformly activating or inhibiting all GPCR‐mediated signals, new strategies focus on selectively directing specific intracellular responses through the use of biased agonists and antagonists, PROTAC‐based modulation, mAbs, nanobodies, aptamers, and gene therapy. These innovative approaches, supported by breakthroughs in structural biology and pharmacology, are expanding the potential of GPCR‐targeted treatments for cancer and other complex diseases, offering improved efficacy and safety over traditional therapies.

### 8.1 Biased Agonists and Antagonists

Approximately 60% of medications in development and 36% of marketed treatments approved by the US FDA target human GPCRs, indicating that GPCRs are an essential target for many medicines. Furthermore, because many GPCRs are implicated in the development and spread of cancer, possibly impacting both treatment effectiveness and patient survival, GPCRs are regarded as some of the most promising targets for treatment in a wide range of solid malignancies. However, only a few GPCRs have been successfully utilized to create medications that block signaling pathways linked to cancer. More knowledge about GPCR biology reveals that functional selectivity and “biased agonism” exist; consequently, the concept of “one medication per GPCR target” is not as prevalent, and more attention is being paid to exploring different pharmacological options.

Understanding the mechanism of GPCR activation is crucial, as it can aid in the development of anticancer medications. There are numerous opportunities to develop innovative cancer treatments. Targeting GPCR signaling with agonists or antagonists and focusing on the unique interactions between GPCRs and their binding partners are two targeted methods for therapeutic development. These approaches aim to deliver anti‐neoplastic medications or toxins directly to malignant cells. For instance, an endocrine treatment for hormone‐responsive prostate cancer that targets the GnRH receptor lowers testosterone levels. This approach helps cure prostate cancer because promoting Prostate cancer proliferation requires the production of testosterone via a signaling pathway that starts with the release of GnRH from the hypothalamus [287]. An immunological method, such as direct vaccination administration, can restrict endogenous agonists from binding to a specific GPCR to achieve the intended neutralizing effect. Immunogen G17DT, a rare example, is being evaluated in a phase III trial to treat pancreatic cancer [288]. The atrasentan and the ETAR antagonist ZD4054 can effectively reduce the proliferation and invasion of cancer cells when combined with the monoclonal HER2‐specific antibody trastuzumab and the EGFR inhibitor gefitinib, respectively. However, atrasentan and ZD4054 are no longer used as anti‐tumor drugs and have been taken out of clinical trials due to their numerous adverse effects on patients [289]. Similar to this, the biased antagonist Vorapaxar can modulate PAR1, which is known to promote tumor invasion and angiogenesis. By blocking G protein activation without affecting β‐arrestin signaling, it potentially reduces thrombotic risks while attenuating malignant behavior in cancers like breast and prostate [290]. Biased ligands are being investigated for supportive care in oncology in addition to their direct antitumor effects. For instance, oliceridine (TRV130), a G protein‐biased μ‐OR agonist, offers potent analgesia with fewer respiratory and gastrointestinal side effects than morphine and may also reduce immunosuppression, which is a significant concern in maintaining immune surveillance during cancer therapy [291].

### 8.2 PROTAC‐Based GPCR Modulation

In targeted protein degradation, PROTACs have revolutionized the field. They provide an innovative approach to modify disease‐relevant proteins, such as GPCRs, in cancer. By recruiting E3 ubiquitin ligases to the target protein, PROTACs induce the selective ubiquitination and subsequent proteasomal degradation of target proteins, in contrast to conventional small‐molecule inhibitors or antagonists that only inhibit receptor activation. Since GPCRs frequently cause tumors through aberrant or biased signaling pathways, this catalytic mode of action enables the complete elimination of both WT and mutant GPCRs from the cell surface [29]. According to recent research, it is feasible to create PROTACs that specifically target GPCRs linked to cancer, such as the estrogen receptor (ER) in breast cancer and the AR in prostate cancer, leading to strong anti‐tumor effects in preclinical models [292]. While most PROTAC development has focused on nuclear hormone receptors and kinases, proof‐of‐concept studies targeting membrane GPCRs are emerging, highlighting challenges such as cell permeability, receptor accessibility, and the selection of appropriate E3 ligases for efficient degradation [293]. Furthermore, in order to overcome compensatory signaling and drug resistance in advanced malignancies, dual‐targeting PROTACs that degrade several oncogenic drivers (such as EGFR and PARP) are being investigated [292]. The clinical translation of GPCR‐targeted PROTACs still faces several challenges, despite these advancements, including improving pharmacokinetics, optimizing ligand affinity, and achieving tissue‐selective degradation to reduce off‐target effects. However, a convincing method to specifically eradicate oncogenic GPCRs and associated pathological signaling networks is provided by the combination of PROTAC technology with the idea of biased GPCR signaling, opening the door for next‐generation cancer treatments.

### 8.3 mAbs Targeting GPCR

GPCRs are the targets of mAbs, which represent an attractive, rapidly evolving frontier in therapeutic development, particularly for cancer and immune‐related diseases. Due to several technical challenges, the development of mAbs against GPCRs has historically lagged behind small‐molecule methods, despite GPCRs being the most prominent family of membrane proteins and representing the targets for a significant portion of approved drugs. GPCRs’ intrinsic instability outside of their native lipid bilayer, their comparatively tiny and frequently conformationally dynamic extracellular regions, and the difficulty in generating high‐quality antigens that maintain the receptor's natural structure and function are some of these challenges [29, 30]. These challenges have begun to be addressed by advancements in structural biology, such as cryo‐electron microscopy, and novel antigen presentation techniques, including proteoliposomes and virus‐like particles, which have enabled the production of antibodies that can recognize native GPCR conformations [30]. By preventing ligand binding, locking the receptor in a specific conformation, or recruiting immune effector functions such as antibody‐dependent cellular cytotoxicity (ADCC), therapeutic mAbs can exert their effects. Interestingly, the defucosylated anti‐CCR4 antibody mogamulizumab is approved for use in cutaneous T‐cell lymphoma and adult T‐cell leukemia/lymphoma, where it facilitates the removal of tumor cells through enhanced ADCC [30, 294]. The therapeutic flexibility of this method is demonstrated by other mAbs, including erenumab, which prevent migraines by targeting the calcitonin gene‐related peptide receptor [295]. mAbs targeting chemokine receptors, such as CXCR4 and CCR5, are being investigated for their potential to prevent metastasis and modulate the TME in the context of cancer. Meanwhile, antibodies targeting aGPCRs, like GPRC5D, are advancing in clinical trials for multiple myeloma [29, 30]. Additionally, recent advancements are making it possible to create bispecific antibodies and ADCs that target GPCRs, thereby expanding the therapeutic options and enabling more accurate targeting of tumor cells. Moreover, structure‐guided antibody engineering allows the production of mAbs that can stabilize GPCRs in particular conformational or signaling states, potentially biasing receptor signaling in a manner advantageous for therapy [29, 30, 31, 32]. Even though challenges like tissue specificity, delivery, and overcoming tumor heterogeneity remain, the combination of advanced antibody engineering, structural insights, and creative antigen design is significantly accelerating the clinical translation of GPCR‐targeted mAbs. mAbs targeting GPCRs are therefore expected to play a significant role in immunotherapy and precision oncology in the years to come.

### 8.4 Emerging Strategies: Nanobody, Aptamers, and Gene Therapy

Emerging strategies for targeting GPCRs in cancer and other diseases are rapidly evolving beyond traditional small molecules and mAbs, with nanobodies, aptamers, and gene therapy offering unique advantages and novel mechanisms of action.

### 8.5 Nanobodies

Single‐domain antibody fragments, known as nanobodies, which are derived from camelid heavy‐chain antibodies, have garnered significant interest due to their small size, high stability, and ability to bind distinct or hidden GPCR epitopes that are often inaccessible to conventional antibodies [296, 297]. Their superior tissue penetration and rapid clearance make them attractive for both diagnostic imaging and targeted therapy [34, 296, 298]. In cancer models, nanobodies have been effectively employed to block GPCR signaling. They can also be engineered as targeting modules for chimeric antigen receptor (CAR)‐T cell therapies, drug conjugates, or photodynamic therapy agents.[34, 298, 299, 300] For instance, in photodynamic therapy, nanobody–photosensitizer conjugates have shown the ability to kill tumor cells that express GPCR selectively, and nanobody‐based CAR‐T cells have shown promise in the treatment of solid tumors [34, 299] By enhancing tumor selectivity and diminishing off‐target effects, nanobody–drug conjugates and nanobody‐functionalized nanoparticles further broaden the therapeutic toolkit [34, 298]

### 8.6 Aptamers

GPCRs can be bound by aptamers, which are short, single‐stranded nucleic acids that fold into particular three‐dimensional structures with great affinity and specificity [33]. Aptamers, selected via the SELEX process, offer several advantages, including reduced immunogenicity, ease of synthesis, and the ability to alter receptor activity either competitively or allosterically [19, 33]. Aptamers have demonstrated inhibitory action that targets GPCRs, such as CCR5 and MRGPRX2. Aptamer–siRNA chimeras have been developed to deliver gene‐silencing payloads to GPCR‐expressing cells with precision, exhibiting potential for both targeted gene therapy and direct receptor inhibition [33]. Additionally, aptamers can be designed to disrupt GPCR dimerization or oligomerization, providing another layer of regulatory control [19, 33, 297].

### 8.7 Gene Therapy

Additionally, gene therapy techniques are advancing, using both viral and nonviral vectors to alter GPCR function or expression at the genetic level. To downregulate oncogenic GPCRs, strategies include delivering genes encoding modified GPCRs, dominant‐negative receptor variants, or gene‐silencing constructs like siRNA or shRNA [301, 302].

### 8.8 Current Clinical Trials Landscape

GPCRs continue to be the most frequently used target class in medicine; 516 approved drugs targeting 121 distinct GPCRs across a wide range of diseases, and 30–50% of marketed drugs act through these receptors [303, 304]. In recent years, there has been a surge in interest in targeting GPCRs for cancer and other complex diseases, even though GPCRs have long been essential to treatments for neurological, metabolic, and cardiovascular disorders, such as bronchodilators for asthma, angiotensin receptor blockers for hypertension, and antipsychotics for mental health [303, 304, 305]. This shift is driven by increasing evidence that GPCRs control key cancer hallmarks, including immune evasion, metastasis, proliferation, and therapy resistance, and that approximately 20% of all malignancies have mutations or abnormal expression of GPCRs [303]. Through clinical trials, several GPCR‐targeted agents are making progress in oncology. Adenosine A2A receptor‐targeting minor molecule antagonist AZD4635 and ONC201, an imipramine that antagonizes the dopamine receptor D2 (DRD2) and has demonstrated efficacy in glioblastoma in phase II trials, are noteworthy examples [303]. Another example is the use of SMO antagonists, which block Hh signaling in basal cell carcinoma and other cancers [303, 305]. Additionally, biologics are gaining momentum. For example, mAbs and nanobodies that target chemokine receptors, such as CXCR4 and CCR4, are being used for metastatic malignancies, as well as novel therapeutic approaches, including peptide‐based medications and ADCs, which are currently undergoing clinical evaluation [305]. GPCR‐targeted medications are still the most common treatment for chronic diseases apart from cancer. While new agents for cardiovascular and respiratory disorders remain in high demand, drugs that target GLP‐1 and GIP receptors, for instance, are transforming the treatment of diabetes and obesity, and are expected to generate approximately $30 billion in sales by 2023 [303, 304]. Allosteric modulators and pathway‐biased ligands are recent clinical developments that aim to optimize GPCR signaling for increased efficacy and fewer side effects in both cancer and noncancer applications [303, 304]. The pipeline remains robust despite challenges, including the structural complexity of GPCRs and the need for more accurate biomarkers. As of 2025, 337 agents targeting 133 GPCRs, including 30 novel targets, were in clinical trials, with a focus on developing novel drug modalities and broadening indications [304]. The landscape of clinical trials for GPCR‐targeted therapeutics is becoming more diverse in terms of drug modalities, disease indications, and mechanistic sophistication. This includes biologics and small compounds targeting immune checkpoints, chemokine receptors, and orphan GPCRs in cancer; however, in other diseases, GPCRs continue to lead the way in drug discovery and development, with an ongoing stream of novel medicines and clinical advancements [303, 306].

In conclusion, the expanding arsenal of GPCR‐targeted therapeutics, including small‐molecule agonists and antagonists, biased ligands, mAbs, nanobodies, aptamers, gene therapies, and novel approaches like PROTACs, reflects the central and multifaceted role of GPCRs in cancer biology and treatment. These strategies exploit the ability of GPCRs to regulate tumor growth, metastasis, immune responses, and the TME, offering new avenues for more selective and effective anticancer treatments. Table 2 summarizes currently utilized anticancer drugs and antibodies targeting GPCRs and their respective targets, highlighting the clinical impact of this receptor family [29, 305, 307].

**TABLE 2:** US FDA‐approved drugs and antibodies currently being used to treat various malignancies.

Drugs	Receptor	Cancer type	Types of drugs	Year of approval	References
Cabergoline	DRD1	Neuroendocrine tumors, pituitary tumors	Small molecule	1996	[308]
Lanreotide	SSTR	Pancreatic cancer	Peptide	2007	[309]
Degarelix	GnRH	Prostate cancer	Peptide	2008	[310]
Plerixafor	CXCR4	Multiple myeloma	Small molecule	2008	[311, 312]
vismodegib (Erivedge)	SMO	Locally advanced and metastatic basal cell carcinoma	Small molecule	2012	[313]
Raloxifene	ER	Breast cancer	Small molecule	2014	[314]
Sonidegib (Odomzo)	SMO	Locally advanced and metastatic basal‐cell carcinoma	Small molecule	2015	[315]
Mogamulizumab	CCR4	Cutaneous/peripheral T‐cell lymphoma; adult T‐cell lymphoma	mAb	2018	[294, 316]
Lutathera (Lutetium Lu 177 dotatate)	SSTR	Gastroenteropancreatic neuroendocrine tumors (GEP‐NETs)	Peptide	2018	[317]
Gilteritinib	Serotonin receptors	Relapsed or refractory acute myeloid leukemia	Small molecule	2018	[318]
Lutetium 177 dotatate	SSTR2	Gastroenteropancreatic neuroendocrine tumors	Radiolabeled peptide	2018	[319]

*Abbreviations*: DRD1: dopamine receptor D1, SSTR: somatostatin receptor, GnRH: gonadotropin‐releasing hormone receptor, CXCR4: C‐X‐C chemokine receptor 4, SMO: smoothened receptor, ER: estrogen receptor, CCR4: C‐C chemokine receptor type 4, mAb: monoclonal antibody.

In cancer and other diseases, combining gene therapy with nanobody or aptamer technologies, such as aptamer‐guided delivery of gene‐editing tools, holds promise for prolonged and selective regulation of GPCR signaling [19, 33]. Collectively, these emerging modalities—nanobodies, aptamers, and gene therapy—are expanding the therapeutic landscape for GPCR‐targeted interventions, offering new avenues for precision medicine, improved selectivity, and the potential to overcome resistance mechanisms associated with conventional therapies [33, 34].

## 9 Preclinical Animal Experiments on Biased GPCR Signaling

Recent years have seen significant advances in understanding biased signaling at GPCRs, particularly through the use of genetically modified mouse models (e.g., β‐arrestin 1 and β‐arrestin 2 knockout mice). These models have enabled the dissection of distinct signaling pathways activated by different ligands, revealing functional selectivity in vivo.

Mice lacking β‐arrestin 1 (β‐arrestin 1–KO mice) were very useful in identifying the potential for developing biased agonists at GPR109a receptors. Niacin (nicotinic acid), which activates GPR109a receptors, has been used for the treatment of cardiovascular disease for many years because it effectively lowers triglyceride levels and raises high‐density lipoprotein levels in the blood [9]. However, its therapeutic use is limited by the side effect of cutaneous flushing. The flushing response induced by niacin is alleviated in mice lacking β‐arrestin‐1 [320]. Moreover, further studies into the mechanism reveal that niacin‐activated GPR109a receptors signal through β‐arrestin 1 to activate phospholipase A2, thereby increasing arachidonic acid levels [321]. Although the cutaneous flushing is attenuated in the β‐arrestin‐1–KO mice, niacin remains efficacious in its ability to reduce serum free fatty acid levels. These in vivo studies suggest that biased ligands that activate GPR109a but do not recruit β‐arrestin‐1 could be helpful in treating dyslipidemia while avoiding the adverse side effect of cutaneous flushing [322]. The question remains, however, whether niacin's actions at GPR109a are the only means it has to lower serum fatty acid levels. A report by Lauring et al. [323] showed that mice lacking GPR109a were still responsive to niacin's lipid‐lowering properties. This underscores the difficulty of determining whether the effects of the agonist are bifurcated at specific signaling points or if there are additional targets that are not being accounted for when working with whole animal systems. Furthermore, the β‐arrestin 2–KO mice have proven to be very useful in identifying examples of functional selectivity in vivo, particularly pathways that utilize β‐arrestin 2 to promote GPCR‐mediated signaling. The first physiological phenotype reported for the β‐arrestin 2–KO mice was their enhanced responsiveness to morphine‐induced antinociception [324, 325], demonstrating a role for β‐arrestin 2 in negatively modulating μ‐OR responsiveness in vivo. Further studies showed that fentanyl and methadone did not reveal enhanced response profiles in the β‐arrestin 2–KO mice [326]. However, fentanyl and methadone were also known to mediate their analgesic effects through the μ‐OR [327]. These early studies suggest that there is a ligand‐directed bias at the MOR, such that the activity of some agonists, including morphine, is more influenced by the presence of β‐arrestin 2 than others. Additional studies using morphine in the β‐arrestin 2–KO mice suggest the contextual importance of receptor signaling. While morphine‐induced antinociception is negatively regulated by β‐arrestins, morphine‐induced constipation, respiratory suppression, and physical dependence appear to involve β‐arrestin 2, as these side effects are significantly diminished in the β‐arrestin 2–KO mice [327, 328, 329]. While these observations may imply a MOR‐β‐arrestin 2‐dependent signaling pathway, such a mechanism has yet to be mechanistically demonstrated in vivo. The development of MOR agonists that are biased toward G protein signaling and against β‐arrestin 2 recruitment may provide valuable tools for ascertaining whether biased signaling underlies the separation of the antinociceptive properties and side effects induced by opiate narcotics. The first opioid agonist identified as a functionally selective agonist is herkinorin [330]. Although it has limited bioavailability, it exhibits antinociceptive effects when injected into the paw of rats in the formalin test, without producing tolerance with repeated dosing [331]. A recently developed biased agonist (TRV130) has shown promising efficacy in pre‐clinical studies. This compound is potent in G protein‐mediated signaling but exhibits minimal efficacy in inducing β‐arrestin 2 recruitment in cell‐based assays. Importantly, in mouse and rat models, TRV130 exhibits analgesic potencies superior to those of morphine, yet it produces less gastrointestinal transit delay and has a lesser effect on respiratory parameters than morphine. TRV130 serves as a proof of concept demonstrating that the effects of an agonist that does not recruit β‐arrestin 2 can recapitulate the behaviors seen in morphine treated mice that lack β‐arrestin 2 and suggest that the development of G protein signaling‐biased MOR agonists may be a means to promote opioid analgesia while limiting specific side effects [291].

Additionally, PAR1 is a member of the four PARs, which also include PAR2‐4, that act as exquisite sensors for a select group of proteases [332]. PAR1 expression is increased in several cancers, including breast, colon, and lung cancer. A study using a xenograft model of breast carcinoma cells initially demonstrated a critical role for MMP‐1 derived from tumor‐infiltrating fibroblasts in the cleavage of PAR1, which appears to drive the migration and invasive behavior of cancer cells [333]. Under pathophysiological conditions in carcinomas, PAR1 is an oncogenic protein, which is a potent inducer of cancer cell migration, invasion, survival, and metastasis [334, 335, 336, 337, 338, 339]. Researchers have evaluated PAR1‐derived PZ‐128 as a potential PAR1 inhibitor to suppress breast cancer progression. PAR1 was topically expressed in PAR1‐null, estrogen‐sensitive MCF‐7 cells and tested for its ability to promote tumor‐growth and invasion in nude mice. PAR1 expression resulted in a 100% rate of tumor formation while PAR1‐null MCF‐7 cells did not form any palpable tumors [333]. These PAR1‐driven tumors could be significantly inhibited (62%, *p* < 0.01) with PZ‐128. PAR1‐dependent tumor growth was mediated by MMP1, as treatment with an MMP‐1 inhibitor resulted in an 82% inhibition of tumor growth. Likewise, PAR1‐expressing MCF‐7 cells formed metastatic lesions in the lungs with a 100% penetrance rate, similar to the highly metastatic MDA‐MB‐231. In contrast, the PAR1‐mutant R310E, which lacked signaling abilities, had undetectable metastatic lesions in the lungs [340]. Most strikingly, there was a similar reduction in the number of metastatic lesions with either treatment as monotherapy, PZ‐128, or MMP‐1 Inh (75 and 88%, respectively, *p* < 0.001) [335]. PAR1 is a poor prognostic marker in lung cancer that correlates with reduced survival in non‐small‐cell lung cancer (NSCLC) [341]. In 2007, bevacizumab (Avastin) was approved in combination with carboplatin and paclitaxel as the first‐line treatment for patients with unresectable, locally advanced, recurrent, or metastatic nonsquamous NSCLC [342]. A comparative efficacy study of PZ‐128 versus Avastin in a well‐established A549 xenograft model of lung adenocarcinoma injected subcutaneously in the flank of the mouse. PZ‐128 and Avastin significantly inhibited tumor growth (*p* < 0.01) with 75 and 67% inhibition, respectively (Table 3) [343]. Moreover the role of MMP‐1 in NSCLC is not well understood. To address the role of mouse homolog MMP‐1a, knockout mice were generated (*MMP1a*–KO) [344]. Lewis lung carcinoma (LLC1) is a highly tumorigenic cell line that expresses high levels of PAR1. Mouse LLC1 cells were implanted subcutaneously into the abdominal fat pad of MMP1a–KO and WT C57CL/6 mice. The progression of tumor growth was monitored over 26 days. The loss of stromal MMP1a resulted in a 50% reduction of tumor growth when compared with WT‐MMP1a mice (*p* < 0.001). There was a significant decrease (30%) (*p* < 0.01) in angiogenesis in the Mmp1a–KO mice when compared with WT mice.

**TABLE 3:** Summary of preclinical studies carried out with PZ‐128 and the MMP1 Inhibitor in breast, lung, and ovarian cancer.

Cell	Tumor model	Type of assay	Outcome	References
MCF‐PAR1	Mammary fat pad NCR nu/nu mice	Xenograft model (tumor size)	>PAR1‐tumor incidence 100% PAR1‐null 0% after 6 weeks	[333]
			>PZ‐128 (10 mg/kg) inhibits 62% MMP‐1 Inh (5 mg/kg) inhibits 82% of tumor growth	
MDA‐MB‐231			>Dual treatment with PZ‐128/taxotere (docetaxel) treatment day 2 98% inhibition	[333]
		Xenograft model (tumor size) TUNEL staining	>Delayed dual treatment day 15 60% inhibition of tumor growth, 60% apoptotic area	[335]
		Western blot	>pAkt as biomarker inhibited 54% by PZ‐128 after 5‐day treatment MMP‐1 Inh 5‐day treatment 61% Inhibition	[335]
MDA‐MB‐231/GFP	Metastasis to lung NCR nu/nu mice, tail vein	Lung histology	>Reduction in metastatic incidence by PZ‐128 75% MMP‐1 Inh 88% inhibition	[335]
			>Reduction in metastatic incidence by PZ‐128 75% MMP‐1 Inh 88% inhibition	[335]
MCF7‐PAR1 (clone N55)			>PAR1‐tumor incidence 100%	[340]
MDA‐MB‐231			MDA‐MB‐231 tumor incidence 100%	[340]
MCF7‐PAR1 (R310E)			R310E tumor incidence 0%	[340]
A549 NCR nu/nu mice	Subcutaneous	Xenograft model tumor size	>75% Inhibition of tumor growth, PZ‐128 monotherapy (10 mg/kg) compared with Avastin (5 mg/kg) 67% inhibition of tumor growth	[343]
OVCAR‐4	Intraperitoneal cavity	Endothelial barrier	>PZ‐128 (10 mg/kg i.p. every other day) reduced ascites formation by 60%	[345]
SKOV‐3			Reduced ascites formation by 60%	[345]
OVCAR‐4	Peritoneal carcinomatosis	Histology	>84–96% inhibition of angiogenesis monotherapy with MMP1 Inh and PZ‐128	[345]
			>Dual treatment with PZ‐128 or MMP‐1 Inh/taxotere (docetaxel) inhibition of metastatic progression through the diaphragm and thoracic cavity	[345]

*Abbreviations*: OVCAR: ovarian cancer cell line 4, SKOV‐3: serous ovarian adenocarcinoma cell line, MCF7: Michigan Cancer Foundation‐7, MDA‐MB‐231: M.D. Anderson—Metastatic Breast Cancer 231.

Ovarian cancer is still one of the deadliest gynecologic malignancies because of early metastatic spread into the abdominal cavity. Despite some advances, a taxane such as docetaxel remains the standard of care in combination with a platinum compound (cisplatin or carboplatin). A murine metastatic model of intraperitoneal (i.p.) ovarian cancer was conducted by injecting extremely aggressive and metastatic OVCAR‐4 cells into the i.p. cavity. All untreated (vehicle‐treated) mice had full‐blown metastasis through the diaphragm and into the lungs and mediastinum (Table 3) [345]. In contrast, dual therapy with PZ‐128 prevented invasion across the diaphragm into the thoracic cavity (*p* = 0.01) compared with docetaxel alone. Similar results were observed with MMP‐1 Inh (*p* = 0.003). These data strongly support an MMP1–PAR1 signaling pathway as an essential target for preventing the metastatic spread in ovarian cancer. Ovarian peritoneal carcinomas produce large volumes of ascitic fluid. Using OVCAR‐4 and SKOV‐3 peritoneal carcinoma models, there was significant inhibition (60%, *p* = 0.0017) of ascitic fluid accumulation using PZ‐128 as monotherapy (10 mg/kg every second day) when compared with vehicle control in an OVCAR‐4 peritoneal carcinoma model. Similar results were seen with SKOV‐3 cells. This effect on ascites accumulation may be a direct consequence of an MMP1–PAR1‐dependent alteration in endothelial barrier function. Taken together, PZ‐128 is an excellent therapeutic for ovarian cancer treatment [345].

In addition to these preclinical advances, several GPCR‐targeted agents have progressed into clinical trials across various cancer types, reflecting their translational potential. Notably, zibotentan and atrasentan, both small‐molecule endothelin A receptor antagonists, are being assessed in prostate cancer across multiple clinical trial phases [305]. In pancreatic and metastatic breast cancer, the Fz receptor‐targeting antibody vantictumab (OMP‐18R5) has shown potential, especially in combination regimens with chemotherapy. Similarly, G17DT, an immunogen targeting the cholecystokinin‐2 receptor, has progressed to phase III trials for pancreatic cancer [346]. In the context of head and neck cancer, vismodegib (GDC‐0449), a SMO receptor inhibitor, is under phase II investigation [347, 348]. Other notable agents include CCX872, a CCR2 antagonist for pancreatic cancer, and mogamulizumab (KW‐0761), an anti‐CCR4 mAb, which is in phase II trials for adult T‐cell leukemia/lymphoma [349]. Several trials also explore the efficacy of beta‐blockers targeting beta‐adrenergic receptors in breast and ovarian cancers, reflecting the utility of drug repurposing [305]. Collectively, these ongoing clinical efforts underscore the expanding landscape of GPCR‐targeted cancer therapeutics and their promise in enhancing precision oncology (Table 4).

**TABLE 4:** Anti‐GPCRs drugs and antibodies under clinical trials.

Cancer	Inhibitor	Type of molecule receptor	Receptor	Phase	References
PCa	Zibotentan (ZD4054) Atrasentan (ABT‐627)	Small molecule	Endothelin A receptor	I, II, III	[350, 351]
HNSCC	GDC‐0449 (vismodegib)	Small molecule	Smoothened receptor (SMO)	II	[347, 348]
OVC	GDC‐044 (vismodegib) Propranolol (β‐blockers)	Small molecule	Endothelin receptor β‐Adrenergic receptor	II, I	[352]
PC	CCX872 (OMP‐18R5) Vantictumab G17DT	Small molecule Antibodies Immunogen	CCR2 Frizzled receptor FZ7 Cholecystokinin‐2 receptor	I, III	[346]
ATCL&L	KW‐0761 (mogamulizumab)	Antibodies	CCR4	II	[349]

*Abbreviations*: PCa: prostate cancer, HNSCC: head and neck cancer, OVC: ovarian cancer, PC: pancreatic cancer, ATCL&L: adult T‐cell leukemia and lymphoma

In summary, preclinical studies have demonstrated that biased GPCR signaling has a significant influence on tumor growth, metastasis, and drug response. Animal models using biased agonists and receptor mutants have provided compelling evidence for the therapeutic advantages of selectively modulating GPCR pathways. Supporting this, multiple GPCR‐targeting drugs are currently in clinical trials for cancers such as prostate, pancreatic, breast, and head and neck. These include endothelin receptor antagonists (zibotentan, atrasentan), Fz and SMO inhibitors (vantictumab, vismodegib), the immunogen G17DT, and repurposed beta‐blockers. These studies highlight the potential of targeting specific GPCR‐mediated signals to enhance efficacy and minimize side effects, thereby paving the way for the translation of biased GPCR modulation into clinical oncology.

## 10 Concluding Remarks and Future Perspectives

GPCRs play a crucial role in cellular communication by initiating multiple signaling pathways that regulate essential functions, including migration, differentiation, growth, and survival. By increasing cell proliferation, encouraging angiogenesis, facilitating invasion and metastasis, and supporting immune evasion, GPCRs aid in the development of cancerous tumors. The understanding of GPCR signaling has evolved recently from a straightforward binary paradigm to a more complex system, in which different GPCR conformations can activate distinct signaling pathways—a process referred to as biased signaling. This advanced understanding creates opportunities for creating cancer treatments that are more efficient and focused. Utilizing the immunomodulatory effects of GPCRs or altering tumor‐immune interactions could transform current immunotherapies to achieve durable clinical responses and prevent tumor recurrence. Research on these combinations is still ongoing, either as a way to treat cancer or as a preventative measure. As our understanding of the complex mechanisms underlying the genesis and spread of cancer advances, GPCRs will play an increasingly significant role as drivers and targets for innovative cancer treatments.

Recent research has clarified the intricate mechanisms behind GPCR‐mediated biased signaling. G protein‐dependent or β‐arrestin‐dependent pathways can be selectively activated by ligand interaction, thereby stabilizing particular GPCR conformations. While it affects multiple stages like tumor invasion, extravasation, and metastatic spread, this selective activation is essential to the development of cancer. To regulate cellular responses, for example, β‐arrestins play a crucial role in coordinating the timing, intensity, and spatial localization of GPCR‐mediated signaling. Furthermore, to predict ligand bias and comprehend the structural dynamics of GPCRs, computational methods such as machine learning models and molecular dynamics simulations have been used. The discovery of biased ligands has been made easier by experimental methods such as mass spectrometry, fluorescence resonance energy transfer, and 19F NMR, which have shed light on the conformational states of GPCRs. Such advances underscore the importance of combining experimental and computational approaches to fully comprehend the complexities of GPCR signaling in cancer.

To minimize side effects and increase therapeutic efficacy, biased ligands that can selectively regulate specific signaling pathways are essential for the future of GPCR‐targeted cancer therapy. Gaining a better knowledge of the structural and conformational dynamics of GPCRs is crucial to achieving this. High‐resolution insights into GPCR structures can be obtained through advancements in structural biology, such as time‐resolved X‐ray crystallography and cryo‐electron microscopy. Additionally, the use of generative AI models, such as GPCR‐BERT, shows potential for creating ligands with desirable bias profiles and predicting receptor conformations. To evaluate the effectiveness and safety of biased ligands, credible biomarkers and patient‐derived models must be developed, as there are still difficulties in transforming these findings into practical applications. Overcoming these obstacles and realizing the potential of biased signaling in cancer treatment will require ongoing interdisciplinary research that integrates structural biology, computational modeling, and pharmacology.

## Author Contributions

I.R.K., S.K., M.A.M., and A.A.B. wrote the manuscript and generated figures. M.A.M. and A.A.B. contributed to the concept and design and critically edited the manuscript. S.A., R.K., A.A.A.S., A.A.B., and M.A.M. critically revised and edited the scientific content. All authors read and approved the final manuscript.

## Conflicts of Interest

The authors declare no conflicts of interest.

## Ethics Statement

The authors have nothing to report.

## Data Availability

The authors have nothing to report.
